# Association of Exposure to Civil Conflict With Maternal Resilience and Maternal and Child Health and Health System Performance in Afghanistan

**DOI:** 10.1001/jamanetworkopen.2019.14819

**Published:** 2019-11-08

**Authors:** Nadia Akseer, Arjumand Rizvi, Zaid Bhatti, Jai K. Das, Karl Everett, Aneesa Arur, Mickey Chopra, Zulfiqar A. Bhutta

**Affiliations:** 1 Centre for Global Child Health, The Hospital for Sick Children, Toronto, Ontario, Canada; 2 Dalla Lana School of Public Health, University of Toronto, Toronto, Ontario, Canada; 3 Center of Excellence in Women and Child Health, Aga Khan University, Karachi, Pakistan; 4 World Bank, Washington, DC

## Abstract

**Importance:**

Current studies examining the effects of Afghanistan’s conflict transition on the performance of health systems, health service delivery, and health outcomes are outdated and small in scale and do not span all essential reproductive, maternal, newborn, and child health interventions.

**Objective:**

To evaluate associations of conflict severity with improvement of health system performance, use of health services, and child nutrition outcomes in Afghanistan during the 2003 to 2018 reconstruction period.

**Design, Setting, and Participants:**

This population-based survey study included a sequential cross-sectional analysis of individual-level panel data across 2 periods (2003-2010 and 2010-2018) and a difference-in-differences design. Surveys included the 2003 to 2004 and 2010 to 2011 Multiple Indicator Cluster Surveys and the 2018 Afghanistan Health Survey. Afghanistan’s 2013 National Nutrition Survey was used to assess nutritional outcomes, and the annual Balanced Scorecard data sets were used to evaluate health system performance. Participants included girls and women aged 12 to 49 years and children younger than 5 years who completed nationally representative household surveys. All analyses were conducted from January 1 through April 30, 2019.

**Exposures:**

Provinces were categorized as experiencing minimal-, moderate-, and severe-intensity conflict using battle-related death data from the Uppsala Conflict Data Program.

**Main Outcomes and Measures:**

Health intervention coverage was examined using 10 standard indicators: contraceptive method (any or modern); antenatal care by a skilled health care professional; facility delivery; skilled birth attendance (SBA); bacille Calmette-Guérin vaccination (BCG); diphtheria, pertussis, and tetanus vaccination (DPT3) or DPT3 plus hepatitis B and poliomyelitis (penta); measles vaccination; care-seeking for acute respiratory infection; oral rehydration therapy for diarrhea; and the Composite Coverage Index. The health system performance was analyzed using the following standard Balanced Scorecard composite domains: client and community, human resources, physical capacity, quality of service provision, management systems, and overall mission. Child stunting, wasting, underweight, and co-occurrence of stunting and wasting were estimated using World Health Organization growth reference cutoffs.

**Results:**

Responses from 64 815 women (mean [SD] age, 31.0 [8.5] years) were analyzed. Provinces with minimal-intensity conflict had greater gains in contraceptive use (mean annual percentage point change [MAPC], 1.3% vs 0.5%; *P* < .001), SBA (MAPC, 2.7% vs 1.5%; *P* = .005), BCG vaccination (MAPC, 3.3% vs −0.5%; *P* = .002), measles vaccination (MAPC, 1.9% vs −1.0%; *P* = .01), and DPT3/penta vaccination (MAPC, 1.0% vs −2.0%; *P* < .001) compared with provinces with moderate- to severe-intensity conflict after controlling for confounders. Provinces with severe-intensity conflict fared significantly worse than those with minimal-intensity conflict in functioning infrastructure (MAPC, −1.6% [95% CI, −2.4% to −0.8%]) and the client background and physical assessment index (MAPC, −1.0% [95% CI, −0.8% to 2.7%]) after adjusting for confounders. Child wasting was significantly worse in districts with greater conflict severity (full adjusted β for association between logarithm of battle-related deaths and wasting, 0.33 [95% CI, 0.01-0.66]; *P* = .04).

**Conclusions and Relevance:**

Associations between conflict and maternal and child health in Afghanistan differed by health care intervention and delivery domain, with several key indicators lagging behind in areas with higher-intensity conflict. These findings may be helpful for planning and prioritizing efforts to reach the United Nations’ Sustainable Development Goals in Afghanistan.

## Key Points

**Question:**

Is conflict severity associated with the performance of health systems and population health outcomes in Afghanistan during the 2003 to 2018 reconstruction period?

**Findings:**

In this survey study of 64 815 women in Afghanistan, notable health and health system improvements were made despite increasing conflict after 2010. However, regions with greater conflict had lower gains in contraceptive use, skilled birth attendance, and child vaccination and increased child wasting, as well as poor health facility infrastructure, client background and physical assessments, and health care professionals’ knowledge level.

**Meaning:**

These findings suggest that efforts to improve contraceptive use, measles vaccination, and functioning health service infrastructure should be prioritized in provinces of Afghanistan that experience severe conflict.

## Teaser

This survey study evaluates associations of conflict severity with improvement of health system performance, use of health services, and child nutrition outcomes in Afghanistan during the 2003 to 2018 reconstruction period.

## Introduction

Humanitarian crises, including war, natural disasters, and population displacement, negatively affect population health and development.^1,2,3,4,5^ Recent analyses have shown significant excess of child mortality in Africa due to conflict,^6^ collapse of health systems, and deterioration of protective infrastructure that further exacerbate mortality and morbidity in conflict settings.^1,2,3^

In the wake of increasing conflict and global instability, the need to understand how to make health systems more resilient and how to effectively shift from acute emergency response to systems building in chronic conflict zones is greater. One approach is to shift service delivery from centralized ministerial bodies to local nongovernmental organizations (NGOs), as in Lebanon^7^ and Afghanistan^8,9,10^ (eMethods 1 in the Supplement).

Afghanistan is a landlocked, impoverished South Central Asian nation of approximately 30 million civilians. For much of the last 4 decades, it has experienced conflict, insurgency, and war. Nonetheless, the resilient nation has made notable gains in developing and rebuilding infrastructure during the past 15 years and has concurrently improved access to and quality of health care service delivery for women and children.^11,12,13,14,15,16^ However, outreach and access challenges remain and are compounded by protracted conflict and its consequences in many provinces.^17^

Several studies of population and health systems resilience in the wake of ongoing conflict in Afghanistan have been performed. Although cross-sectional and analytical studies exist,^14,15,18,19,20,21,22,23,24,25,26,27,28,29,30^ they have had limited scope, and few assessed the continuum of reproductive, maternal, and child health. We undertook a longitudinal assessment of Afghanistan’s entire redevelopment period (2003-2018) using individual-level data sets from robust national survey data to further explore these gaps. We specifically assessed associations between ongoing conflict in Afghanistan and the levels and progress in maternal and child health service coverage, performance of health systems, and child nutrition during the 2003 to 2018 reconstruction period.

## Methods

### Study Design and Approach

This survey study used sequential panel data sets from large representative surveys to examine progress and trends during 2 critical transitionary periods in Afghanistan: the 2003 to 2010 immediate post-Taliban period when the bulk of reconstruction and development took place, and the 2010 to 2018 period, when the health financing models and security context changed. The association between changing levels of conflict and outcomes was examined at the ecological level (across study periods), at the macro level (across provinces and districts), and at the micro level (across households and health facilities). The protocol for the study was approved by the Aga Khan University ethics review committee, and waiver was obtained for the quantitative analysis of publicly available data sets. All participants provided written or oral informed consent. This study followed the Strengthening the Reporting of Observational Studies in Epidemiology (STROBE) reporting guideline.

### Key Data Sources

We vetted all good-quality representative population-based health, nutrition, and health systems surveys conducted in Afghanistan after 2001. Quality assessment of relevant maternal and child health surveys in Afghanistan was conducted in an earlier study.^12^ Surveys that aligned with our study periods, had adequate population coverage, and used standardized methods were considered in our analyses. For evaluation of reproductive, maternal, newborn, and child health service coverage, we used the 2003-2004 and 2010-2011 Multiple Indicator Cluster Surveys^31,32^ and the 2018 Afghanistan Health Survey.^33^ The surveys informed analyses for the 2003 to 2010 and 2010 to 2018 periods. Child nutrition data were obtained from the Afghanistan 2013 National Nutrition Survey.^34^ The National Nutrition Survey was a large-scale nationally and provincially representative survey that collected population data on anthropometry, infant and young child feeding, and micronutrient deficiencies. The original individual or household was analyzed for all surveys, and available sample sizes are detailed in eTable 1 in the Supplement. For the Multiple Indicator Cluster Surveys and Afghanistan Health Survey, the purpose and content of the interviews were outlined to every participant, and informed consent was sought before any data collection.

Indicators of health system performance were analyzed from the Afghanistan Balanced Scorecards (BSC) data sets, a series of annual (2004-2016) performance assessments of 6 key health service domains, consisting of 23 indicators and 3 summary indicators.^35^ Each indicator is scored from 100, with higher scores indicating better performance. The sampling frame for the BSC consists of a random sample of patients and health workers from as many as 25 randomly selected comprehensive health centers, basic health centers, and health subcenters in each of the 34 provinces. Data are collected by surveyors from Kabul in more secure provinces and by trained local school teachers in less secure provinces.

Although BSC indicator definitions changed over time, generally, the 2004 to 2010 definitions are comparable, and so were the 2011 to 2016 definitions. For 2004 to 2016 annual survey rounds, data were available on 617 to 783 health facilities, 5719 to 7979 patient–health care professional interactions, 5597 to 7979 patient exit interviews, and 1452 to 2520 health worker interviews.^36^ We analyzed all original BSC data and reconstructed indicators of health facility performance. An overview of all data sources used in this analysis is provided in eTable 2 in the Supplement.

### Conflict (Exposure) Definition

Data on conflict and insecurity in Afghanistan were obtained from the Uppsala Conflict Data Program,^37^ which has existed since 1970 and is the most frequently cited global source on armed conflict data. We obtained battle-related deaths (BRDs; defined as the use of armed force between warring parties in a conflict dyad, state based or non–state based, resulting in deaths [eg, civilian, forces, etc]) by encounter and generated totals for Afghanistan’s 34 provinces from 2003 to 2017. We adapted the conflict intensity classification used by the Uppsala Conflict Data Program^37^ to fit the study periods and desired levels of conflict for this analysis. Provinces were grouped into minimal-, moderate-, and severe-intensity conflict zones by study periods (2003-2010 and 2010-2017). Provinces with severe-intensity conflict had at least 1000 BRDs in any 3 consecutive years; with moderate-intensity conflict, 300 to 1000 total BRDs in any 3 consecutive years; and with minimal-intensity conflict, less than 300 total BRDs in any 3 consecutive years.

We explored alternative classifications of conflict using measures of perception regarding fear collected as part of the 2018 Survey of the Afghan People^38^ and the United Nations reports on security in Afghanistan^39^ but ultimately decided that BRDs were a more objective measure. Further detail on our conflict classification is included in eMethods 2 in the Supplement.

### Outcome Variables

Reproductive, maternal, newborn, and child health interventions coverage was examined using 10 standard indictors that span the continuum of care: contraceptive method (any or modern); antenatal care (ANC) by a skilled health care professional; facility delivery; skilled birth attendance (SBA); bacille Calmette-Guérin vaccination (BCG); diphtheria, pertussis, and tetanus vaccination (DPT3); measles vaccination; care-seeking for acute respiratory infection; oral rehydration therapy for diarrhea; and the Composite Coverage Index (CCI). The CCI is a commonly used composite of overall health coverage that includes curative and preventative child and maternal health interventions.^40^ Domains and component indicators are defined in eTable 3 in the Supplement.

Performance of the health system was analyzed using standard BSC composite domains (client and community, human resources, physical capacity, quality of service provision, management systems, and overall mission)^35^ and additional key component indicators as deemed relevant to health facility access and capacity in Afghanistan. We examined absolute mean differences for 6 broad health systems domains and an overall health system performance composite measure. Domains and component indicators are defined in eTable 4 in the Supplement.

Child anthropometry indicators were examined as nutritional outcomes. We calculated *z* scores for height for age, weight for age, and weight for height according to World Health Organization child growth standards^41^ for children younger than 5 years. Children more than 2 SD below the median of the World Health Organization reference population for height for age were categorized as stunted; more than 2 SD below the median of the reference population for weight for age, as underweight; or more than 2 SD below the median of the reference population for weight for height, as wasted. We also calculated the joint probability of stunting and wasting to examine concurrent malnutrition among Afghan children.

### Statistical Analysis

To examine the association between conflict and child nutrition outcomes, we conducted a series of stepwise multivariable linear regression models using district-level (399 districts) data. Details are provided in eMethods 2 in the Supplement.^42,43,44^ Univariate statistics were estimated using means (SDs) and frequencies or proportions as appropriate. We calculated mean differences, differences of mean differences, and annual percentage point changes in outcomes. We used 2-sided *t* tests and 1-way analysis of variance methods with ad hoc Tukey comparisons (type I error rate constrained at .05) to examine statistical differences across conflict subgroups.

To understand the association of conflict with the use of health services and health system performance outcomes, the cross-sectional surveys were assembled into panel data sets and the difference-in-differences (DID) analysis methods were used.^45^ We withheld the assumption of parallel trends for all analyses (ie, absence of conflict would result in the mean outcomes of conflict and nonconflict provinces to follow parallel paths over time). In addition, we used interaction estimators in unadjusted and covariate-adjusted regression methods to estimate the DID effect. The general model specification included an interaction term between study period (categorical or continuous) and conflict dummy variables (moderate or severe intensity). Control variables were included as fixed or time-variant confounders measured at the facility or the province level. Generalized linear models and generalized estimating equations were fitted through xtreg and xtgee routines, respectively, in Stata software, version 14 (StataCorp LLC). The reproductive, maternal, newborn, and child health intervention outcome models were adjusted for percentage of female illiteracy, rural population, and the type of NGO-contracting mechanism operating in the province (contracting in or out). The health system performance models were adjusted for patient volume, facility type (basic, comprehensive, or subcenter), NGO-contracting mechanism, and geographic region. Although a range of potential confounders were identified from the literature and expert opinion (eg, poverty, mean distance to city center, and distance to Kabul city), the above variables were included for parsimony and to avoid overfitting models. Effect estimates were reported with 95% CIs, and statistical significance was held at 2-sided *P* < .05 and *P* < .10 for borderline significance. All analyses considered the survey design and weighting characteristics except pooled analyses, because design and weighting parameters differed between surveys. Statistical analyses were conducted from January 1 through April 30, 2019, using Stata software, version 14 (StataCorp LLC).

## Results

Responses from a total of 64 815 women across surveys (mean [SD] age, 31.0 [8.5] years) were analyzed. By 2018, 73.4% of women lived in areas of moderate- or severe-intensity conflict compared with only 26.6% in 2003.

### Insecurity Change Over Time

**Figure 1.**
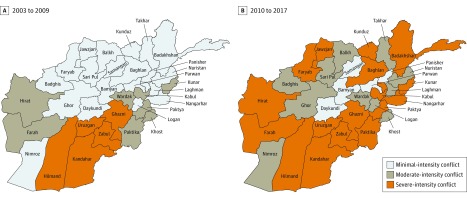
Conflict Intensity in Provinces of Afghanistan

Conflict increased across regions of Afghanistan from 2003 to 2018 (Figure 1). From 2003 to 2010, 21 of 34 provinces (61.8%) had minimal-intensity conflict, 8 (23.5%) had moderate-intensity conflict, and 5 (14.7%) had severe-intensity conflict. From 2010 to 2018, conflict escalated in several provinces, shifting the distribution toward 5 (14.7%) with minimal-intensity, 11 (32.4%) with moderate-intensity, and 17 (50.0%) with severe-intensity conflict. Battle-related deaths by province and year are provided in eTable 5 in the Supplement.

### Use of Health Services and Conflict

**Figure 2.**
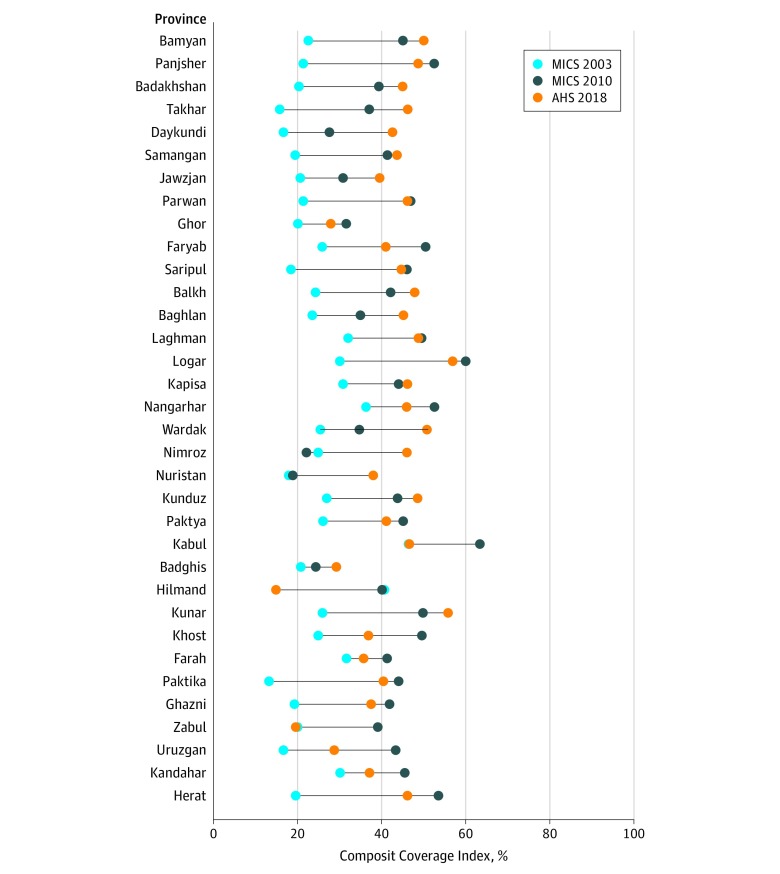
Composite Coverage Index Levels and Change From 2003 to 2018 by Province The Composite Coverage Index indicates overall health coverage, including curative and preventative child and maternal health interventions. Provinces are listed in descending order of total battle-related deaths from 2003 to 2009. The vertical lines represent percentage point changes between years. AHS 2018 indicates 2018 Afghanistan Health Survey; MICS 2003 and 2010, the 2003-2004 and 2010-2011 Multiple Indicator Cluster Surveys.

The CCI improved in all but 2 provinces in both study periods, although coverage levels remained suboptimal overall, with only 4 of 34 provinces having coverage greater than 50% in 2018 (Figure 2), and changes in the use of health services varied by domain. From 2003 to 2010, no clear association was evident between the province’s BRDs and mean annual percentage point change (MAPC) in the CCI (eFigure 1A in the Supplement). In 2010 to 2018, provinces with higher levels of conflict generally had less CCI gain, although variation existed across provinces. Severe-intensity conflict provinces (>4000 BRDs [Uruzgan, Nangarhar, Kandahar, and Hilmand]) regressed (MAPC, −3.7%) (eFigure 1B in the Supplement).

Progress in crude (absolute) changes for specific interventions by conflict status in 2003 to 2010 is shown in eTable 6, eTable 7A, and eFigure 2 in the Supplement. After controlling for confounders (literacy, health service–contracting mechanism, and rural residence), provinces with moderate-intensity conflict had greater gains in contraceptive use (DID β = 1.03 [95% CI, 0.73-1.32]), ANC (DID β = 0.43 [95% CI, 0.06-0.80]), SBA (DID β = 0.53 [95% CI, 0.20-0.86]), and care seeking for acute respiratory infection (DID β = 0.59 [95% CI, 0.16-1.02]) compared with more secure provinces (Table 1). The rates of improvement between conflict groups were often very different. For example, contraceptive use improved per year at a mean of 2.2% vs 0.2% for severe- vs minimal-intensity conflict provinces (*P* < .001). Childhood vaccination coverage declined in severe- compared with minimal-intensity conflict provinces after controlling for confounders, including BCG (MAPC, −0.5% vs 3.3%; *P* = .002), DPT3/penta (MAPC, −2.0% vs 1.0%; *P* < .001), and measles (MAPC, −1.0% vs 1.9%; *P* = .01) (eTable 8 in the Supplement). Full model results are included in eFigure 2, eTable 6, and eTable 8 in the Supplement.

**Table 1.** Adjusted DID Associations Between Conflict Severity and Key Reproductive, Maternal, Newborn, and Child Health Interventions^a^

DID Estimator by Conflict Intensity × Time^b^	MICS 2003 to MICS 2010		MICS 2010 to AHS 2018	
	β Coefficient (95% CI)	*P* Value	β Coefficient (95% CI)	*P* Value
Contraceptive use				
Moderate-intensity conflict × time	1.03 (0.73 to 1.32)	<.001	–0.85 (–1.14 to –0.57)	<.001
Severe-intensity conflict × time	1.22 (0.74 to 1.69)	<.001	–0.50 (–0.92 to –0.08)	.02
Antenatal care				
Moderate-intensity conflict × time	0.43 (0.06 to 0.80)	.02	–0.63 (–0.97 to –0.30)	<.001
Severe-intensity conflict × time	0.66 (–0.05 to 1.37)	.07	–0.01 (–0.59 to 0.57)	.97
Skilled birth attendant				
Moderate-intensity conflict × time	0.53 (0.20 to 0.86)	.002	–0.53 (–0.89 to –0.16)	.005
Severe-intensity conflict × time	0.43 (–0.38 to 1.24)	.29	0.10 (–0.55 to 0.75)	.76
BCG vaccine				
Moderate-intensity conflict × time	0.05 (–0.45 to 0.55)	.85	–1.30 (–1.79 to –0.81)	<.001
Severe-intensity conflict × time	–1.33 (–2.17 to –0.49)	.002	0.06 (–0.75 to 0.88)	.88
DPT3/penta vaccine				
Moderate-intensity conflict × time	–0.07 (–0.51 to 0.38)	.77	–0.88 (–1.33 to –0.43)	<.001
Severe-intensity conflict × time	–2.11 (–3.05 to –1.17)	<.001	1.56 (0.71 to 2.41)	<.001
Measles vaccine				
Moderate-intensity conflict × time	0.47 (0.01 to 0.94)	.04	–0.30 (–0.74 to 0.14)	.44
Severe-intensity conflict × time	–1.15 (–2.03 to –0.27)	.01	0.33 (–0.51 to 1.17)	<.001
Oral rehydration therapy				
Moderate-intensity conflict × time	–0.43 (–0.97 to 0.10)	.11	0.03 (–0.41 to 0.48)	.89
Severe-intensity conflict × time	2.78 (2.06 to 3.50)	<.001	–0.97 (–1.63 to –0.31)	.004
Care seeking for acute respiratory infection				
Moderate conflict × time	0.59 (0.16 to 1.02)	.008	–0.03 (–0.51 to 0.45)	.91
Severe conflict × time	–0.30 (–1.29 to 0.69)	.56	0.12 (–0.88 to 1.12)	.81

Abbreviations: AHS, Afghanistan Health Survey; DID, difference-in-difference; DPT3, diphtheria, pertussis, and tetanus (3 doses); MICS, Multiple Indicator Cluster Survey; penta, DTP3 plus hepatitis B and poliomyelitis.

^a^ Models were adjusted for the main effects of conflict and time and covariates including maternal illiteracy, contracting type, and rural residence. Complete results are given in the eFigure 2 and eTable 6 in the Supplement.

^b^ Minimal-intensity conflict is the reference category.

Progress in crude (absolute) changes in specific interventions by conflict group in 2010 to 2018 are also shown in eTable 6, eTable 7B, and eFigure 2 in the Supplement. After controlling for confounders, provinces with severe- to moderate-intensity conflict had slower gains in contraceptive use (β = −0.85 [95% CI, −1.14 to −0.57]), SBA (β = −0.53 [95% CI, −0.89 to −0.16]), DPT3 vaccination (β = −0.88 [95% CI, −1.33 to −0.43]), and BCG vaccination (β = −1.30 [95% CI, −1.79 to −0.81]) relative to provinces with minimal-intensity conflict (*P* < .001) (eTable 8 in the Supplement and Table 1). Provinces with minimal-intensity conflict had greater gains in contraceptive use (MAPC, 1.3% vs 0.5%; *P* < .001) and SBA (MAPC, 2.7% vs 1.5%; *P* = .005) compared with provinces with moderate or severe conflict after controlling for confounders. Use of DPT3 improved faster in severe- relative to minimal-intensity conflict provinces (MAPC, 6.1% vs 4.3%; *P* < .001), although they also started at lower baseline levels in 2010 (6.2% vs 38.0% coverage). Use of ANC and oral rehydration therapy regressed from 2010 to 2018, irrespective of conflict severity. Crude box plots, complete regression models, and covariate-adjusted prevalence plots for each indicator over time are shown in eTable 6 and eFigure 2 in the Supplement.

### Health Systems Performance and Conflict

Overall health system performance (as measured by the composite score across several domains) improved for most provinces in 2004 to 2010 (absolute gain range, 1.3%-25.5% [excluding Zabul]) (eFigure 3A in the Supplement) and in 2011 to 2016 (range, 0.1%-48.3% [excluding Kapisa, Kunar, and Badghis, which regressed slightly]) (eFigure 3B in the Supplement). Descriptively, the overall score for health system performance did not show any clear association with BRDs in 2004 to 2010 or 2011 to 2016 (eFigure 4 in the Supplement).

Crude results stratified by conflict status for 2004 to 2010 are presented in eTable 9A in the Supplement. After adjusting for confounders (ie, patient volume, type of health facility, geographic region, and contracting mechanism) (Table 2), equipment functionality deteriorated at 3.3% (95% CI, −5.1% to −1.5%) annually among health facilities in provinces with moderate-intensity conflict and 12.4% (95% CI, −21.4% to −3.4%) per year among provinces with severe-intensity conflict compared with provinces with minimal-intensity conflict.

**Table 2.** Adjusted DID Estimation of Associations Between Conflict Severity and Key Health Systems Indicators^a^

DID Estimator by Conflict Intensity × Time^b^	2004-2010		2011-2016	
	β Coefficient (95% CI)	*P* Value	β Coefficient (95% CI)	*P* Value
Equipment functionality index				
Moderate-intensity conflict × time	–3.3 (–5.1 to –1.5)	<.001	0.1 (–0.4 to 0.6)	.59
Severe-intensity conflict × time	–12.4 (–21.4 to –3.4)	.01	–0.4 (–0.9 to 0.1)	.11
Drug availability index				
Moderate-intensity conflict × time	0.2 (–2.8 to –3.1)	.90	NA	NA
Severe-intensity conflict × time	13.8 (–1.0 to 28.6)	.07	NA	NA
Pharmaceuticals and vaccines availability index				
Moderate-intensity conflict × time	NA	NA	1.9 (1.3 to 2.4)	<.001
Severe-intensity conflict × time	NA	NA	1.5 (0.9 to 2.0)	<.001
Infrastructure index				
Moderate-intensity conflict × time	–2.3 (–4.8 to 0.2)	.08	NA	NA
Severe-intensity conflict × time	7.0 (–5.4 to 19.4)	.27	NA	NA
Functional infrastructure index				
Moderate-intensity conflict × time	NA	NA	–0.4 (–1.2 to 0.5)	.41
Severe-intensity conflict × time	NA	NA	–1.6 (–2.4 to –0.8)	<.001
Patient history and physical examination index				
Moderate-intensity conflict × time	–1.0 (–0.8 to 2.7)	.27	NA	NA
Severe-intensity conflict × time	–4.0 (–12.8 to 4.8)	.37	NA	NA
Client background and physical assessment index				
Moderate-intensity conflict × time	NA	NA	0.4 (–0.2 to 0.9)	.18
Severe-intensity conflict × time	NA	NA	–1.0 (–1.5 to –0.5)	<.001
Patient counseling index				
Moderate-intensity conflict × time	–0.9 (–3.4 to –1.6)	.47	NA	NA
Severe-intensity conflict × time	6.9 (–5.8 to –19.5)	.29	NA	NA
Client counseling index				
Moderate-intensity conflict × time	NA	NA	2.2 (1.4 to 3.1)	<.001
Severe-intensity conflict × time	NA	NA	0.1 (–0.7 to 0.9)	.73
Female health worker index				
Moderate-intensity conflict × time	NA	NA	0.4 (–0.3 to 1.1)	.23
Severe-intensity conflict × time	NA	NA	0.2 (–0.4 to 0.8)	.48
Health care professional knowledge score				
Moderate-intensity conflict × time	NA	NA	1.0 (0.3 to 1.6)	.003
Severe-intensity conflict × time	NA	NA	1.2 (0.6 to 1.8)	<.001

Abbreviations: DID, difference-in-difference; NA, not available.

^a^ Models are adjusted for the main effects of conflict and time and covariates including patient volume, facility type, geographic region, and contracting type; complete results are given in eFigure 5 and eTable 10 in the Supplement.

^b^ Time is a binary indicator for the years at the beginning and end of each period. The interaction term indicates whether the change in the covariate was associated with a change in the outcome over time. Minimal-intensity conflict × time is the reference category.

Crude results stratified by conflict status for 2011 to 2016 are presented in eTable 9B in the Supplement. After adjusting for confounders, provinces with severe-intensity conflict had less gain in functioning infrastructure (MAPC, −1.6% [95% CI, −2.4% to −0.8%]) and the client background and physical assessment index (MAPC, −1.0% [95% CI, −0.8% to 2.7%]) when compared with provinces with minimal-intensity conflict, whereas provinces with moderate-intensity conflict had greater improvement in client counseling index (2.2% per year [95% CI, 1.4%-3.1%]), health care professional knowledge score (1.2% per year [95% CI, 0.6%-1.8%]), and pharmaceuticals and vaccine availability index (1.9% per year [95% CI, 1.3%-2.4%]) (Table 2). Crude box plots, adjusted means, and complete regression models are included in eFigure 5 and eTable 10 in the Supplement.

### Child Nutrition Outcomes and Conflict

Table 3 displays results of the stepwise multivariable regression of child nutritional outcomes on lagged conflict (logarithm 4-year BRDs). In crude analysis, conflict was positively associated with increased wasting prevalence (β = 0.60 [95% CI, 0.25-0.96]). This association remained statistically significant even after controlling for household poverty and maternal illiteracy (β = 0.62 [95% CI, 0.28-0.95]), and further for improved household water and sanitation environment (β = 0.33 [95% CI, 0.01-0.66]), although the effect attenuated notably after the latter adjustments. In terms of relative importance, conflict ranked about as important as maternal illiteracy (standardized βs, 0.09 and 0.11, respectively) as a correlate of wasting prevalence, and household poverty and lack of improved sanitation generally had very strong contributions to wasting (standardized βs, 0.30 and 0.29, respectively). No other child nutrition outcome was significantly associated with conflict. Of note, child stunting had an inverse association with conflict (ie, more conflict was associated with decreased stunting), which was statistically significant (β = −0.71 [95% CI, −1.38 to −0.03]) in the fully adjusted model.

**Table 3.** Crude and Adjusted Association of Conflict With Child Nutritional Outcomes

Outcomes	Model 1^a^			Model 2^b^			Model 3^c^		
	β Coefficient (95% CI)	*P* Value	Standardized β	β Coefficient (95% CI)	*P* Value	Standardized β	β Coefficient (95% CI)	*P* Value	Standardized β
**Wasting**									
Conflict-related deaths, logarithm of 4-y counts	0.60 (0.25 to 0.96)	.001	0.17	0.62 (0.28 to 0.95)	<.001	0.17	0.33 (0.01 to 0.66)	.04	0.09
Household poverty, % poorest 2 quintiles	NA	NA	NA	0.01 (–0.01 to 0.04)	.30	0.05	0.07 (0.04 to 0.10)	<.001	0.30
Maternal illiteracy	NA	NA	NA	0.11 (0.07 to 0.14)	<.001	0.31	0.04 (0.00 to 0.08)	.05	0.11
Household unimproved									
Water	NA	NA	NA	NA	NA	NA	0.04 (0.02 to 0.07)	.001	0.18
Sanitation	NA	NA	NA	NA	NA	NA	0.09 (0.06 to 0.12)	<.001	0.29
**Stunting**									
Conflict-related deaths, logarithm of 4-y counts	–0.61 (–1.54 to 0.31)	.19	–0.06	–0.50 (–1.16 to 0.16)	.14	–0.05	–0.71 (–1.38 to –0.03)	.04	–0.07
Household poverty, % poorest 2 quintiles	NA	NA	NA	0.11 (0.06 to 0.16)	<.001	0.18	0.16 (0.09 to 0.22)	<.001	0.25
Maternal illiteracy	NA	NA	NA	0.53 (0.46 to 0.59)	<.001	0.59	0.47 (0.39 to 0.55)	<.001	0.53
Household unimproved									
Water	NA	NA	NA	NA	NA	NA	0.04 (–0.01 to 0.09)	.11	0.07
Sanitation	NA	NA	NA	NA	NA	NA	0.06 (–0.01 to 0.13)	.10	0.07
**Underweight**									
Conflict-related deaths, logarithm of 4-y counts	–0.08 (–0.72 to 0.56)	.81	–0.01	–0.01 (–0.53 to 0.51)	.98	0.00	–0.27 (–0.79 to 0.25)	.31	–0.04
Household poverty, % poorest 2 quintiles	NA	NA	NA	0.07 (0.03 to 0.11)	<.001	0.17	0.13 (0.09 to 0.18)	<.001	0.31
Maternal illiteracy	NA	NA	NA	0.29 (0.24 to 0.35)	<.001	0.49	0.22 (0.16 to 0.29)	<.001	0.37
Household unimproved									
Water	NA	NA	NA	NA	NA	NA	0.06 (0.02 to 0.10)	.002	0.15
Sanitation	NA	NA	NA	NA	NA	NA	0.07 (0.02 to 0.12)	.009	0.13
**Stunting and Wasting**									
Conflict-related deaths, logarithm of 4-y counts	0.01 (–0.10 to 0.12)	.91	0.01	0.01 (–0.09 to 0.12)	.77	0.01	–0.02 (–0.13 to 0.09)	.67	–0.02
Household poverty, % poorest 2 quintiles	NA	NA	NA	0.01 (0.00 to 0.02)	.03	0.12	0.02 (0.01 to 0.03)	<.001	0.25
Maternal illiteracy	NA	NA	NA	0.01 (–0.00 to 0.02)	.06	0.10	0.00 (–0.02 to 0.01)	.65	–0.03
Household unimproved									
Water	NA	NA	NA	NA	NA	NA	0.01 (0.01 to 0.02)	<.001	0.21
Sanitation	NA	NA	NA	NA	NA	NA	0.00 (–0.00 to 0.02)	.26	0.07

Abbreviation: NA, not applicable

^a^ Indicates crude association.

^b^ Adjusted for household poverty (percentage of population in the poorest 2 quintiles) and maternal illiteracy.

^c^ Further adjusted for household lack of access to improved water and sanitation sources.

## Discussion

Several important findings emerge from our analysis. First, despite widespread conflict, Afghanistan has made significant progress in addressing key population needs for maternal and child health. These gains are notable across a range of indicators reflective of health system performance, community- and individual-level behaviors, and care seeking. Second, security has rapidly deteriorated nationwide in recent years and has resulted in plateauing and even regression of gains in health and use of services. Health interventions particularly at risk include contraceptive use, ANC, SBA coverage, oral rehydration therapy, and measles and BCG vaccination rates. Health facility infrastructure, client evaluations, and health care professional knowledge also worsened in recent years. The ongoing conflict and insecurity has severely affected child nutrition, specifically with an increase in child wasting in the most insecure districts.

Afghanistan’s progress despite ongoing insecurity is likely reflective of targeted donor actions and the NGO-led delivery model for scaling up health services in hitherto unreachable parts of the country^9,32,44,46^ as well as investments in oversight and the supply chain.^20^ Our narrative of Afghanistan’s resilience in the wake of ongoing insecurity and skilled worker and infrastructure shortages is similar to that of previous literature in Afghanistan and other conflict-affected settings.^9,18,19,20,21,22,23,24,25,26,27,28,46,47,48,49^ With the government’s establishment of a basic package of services and delivery through NGO-contracting mechanisms (contracting out as well as contracting in), Afghanistan was able to scale up a combination of primary and secondary care services addressing maternal, newborn, and child health and communicable diseases.^9^ These choices were made early on, given the unique challenges and shortages of human resources in most areas. The recent depreciation of coverage and its effects appear to be linked to worsening conflict and insecurity in our analysis and could also be indicative of poor quality of care across various tiers of the health system.

The slower progress in delivery of essential child vaccines (including measles, BCG, and DPT3) in provinces with moderate- and severe-intensity conflict, despite successive national immunization campaigns and catch-up rounds such as for measles in 2001 to 2002, 2003, 2006 to 2007, and 2009,^50^ could reflect the time taken to establish the immunization system, mobile health teams, and community-based health care programs,^11,51^ which were not yet established in Afghanistan in the early years of the study period (2003-2005). The slow progress could also reflect potential misuse of resources, particularly those allocated for public health campaigns. Such fund mismanagement and poor oversight continues to be a challenge in Afghanistan.^52^ In the early years of redevelopment, health facilities in insecure provinces also lacked in equipment functionality and physical infrastructure, which could be associated with practical and logistical challenges in delivering, developing, and maintaining large-scale health facilities in such areas. Our results suggest that recent resurgence in conflict is negatively associated with health and systems gains in Afghanistan, particularly in extreme conflict zones such as Kandahar, Helmand, Zabul, and Urozgan. Several community- and facility-based interventions are at risk. Our finding that key maternal indicators such as ANC and SBA, which require a functional health system, are among the first to fall behind is not surprising. These indicators are among the most inequitable in low- to middle-income countries and particularly so in conflict settings.^53^ Family planning interventions are strongly linked to culture and religious belief in Afghanistan; thus, poor uptake in higher-conflict areas could suggest heightened physical and cultural barriers given conservative ideologies among warring factions in those regions.^54^ The pathways linking increased conflict and insecurity to poor health facility infrastructure, client evaluations, and knowledge of health care professionals that were identified in our study require further investigation. Our findings that child emaciation increased with greater insecurity are similar to the findings of studies in other unstable and emergency contexts,^55,56,57,58^ including assessments of recent conflicts in Somalia^55^ and Nigeria,^56^ and suggest that acute food deprivation is a continued challenge that requires immediate attention in conflicted provinces of Afghanistan.

### Implications for Afghanistan

Afghanistan has had a successful model of contracting out health services to NGOs (eMethods 1 in the Supplement), which has effectively improved access despite security issues.^11,14^ It appears that improving on and sustaining this model, particularly with the inclusive role of community health workers, will be a critical strategy to target the facility- and community-based health care interventions that are at risk of regressing in areas with severe-intensity conflict.^13^ The community health workforce has played a critical role in overcoming health worker shortages in low- to middle-income countries, particularly in hard-to-access and insecure areas, and has been linked to notable improvements in maternal and child health in South Asia.^59,60^

Strengthening the capacity of existing health facilities and quality of care is essential to further gains. Particular focus should be given to ensuring functional and sound facility infrastructure, appropriate assessment of patients, and improving the knowledge of health care professionals. Availability and accessibility can be improved by building on the existing health service delivery mechanisms in the country. Health care staff, such as physicians, nurses, vaccinators, and technicians, have also fallen behind population needs, which may be associated with the observed reversal of gains found in our study; improvements should be urgently prioritized by the government.^18^ Across all cadres, culturally appropriate efforts to encourage and attract support for female community health workers in remote public health settings are needed. Particularly in conflict areas, such initiatives could encourage the use and acceptability of family planning interventions. Emergency medicine is one area of health care that is not considered in the basic package of health services and national health policies in Afghanistan^9,27^ but should be included, given increasing conflict. Government and development partners must focus on peace and reconciliation efforts. Mobilizing community Shuras (consultative councils) to mediate among government, NGOs, and armed oppositions groups has been tentatively successful in Afghanistan, although perhaps not sustainable.^24^

### Strengths and Limitations

Several limitations should be recognized in considering these findings. Given the lack of a reliable district health management information system and a formal mechanism for civic registration and vital statistics,^61^ we mainly relied on available household survey data. The BSC provided standardized annual data on health facilities; however, no linkage of facilities to household data in catchment populations was available, thus limiting inferences about population. Despite this, our unique pseudocohort approach to linking several good-quality, large-scale surveys on health and the health system in Afghanistan is a major strength of this study; it permitted otherwise diverse cross-sectional surveys to be analyzed concurrently with a narrative of a comprehensive story of change across decades.

We relied on Uppsala’s BRDs as a means of classifying the severity of conflict, but we recognize this data set’s limited ability to represent the full dimensions of conflict and insecurity. Nonetheless, we examined several classifications of conflict severity using BRDs in sensitivity analyses with consistent findings, which strengthens our results.

Future data collection efforts should attempt to collect and triangulate household data to assess resilience and the effect of insecurity. For example, the 2017 Survey of the Afghan People collected nationally and provincially representative civilian perceptions on conflict and insecurity and their effect on household experiences and decision-making.^38^

Our multivariable quantitative analysis used the increasingly common and powerful DID methods to control for baseline values and secular trends in outcomes when assessing the association of an environmental shock (ie, conflict).^62^ We also have large sample sizes from panel cross-sectional surveys, which strengthened statistical power and inferences. Nonetheless, a few limitations should be noted. The parallel trends assumption was difficult to assess because pre-2003 data in Afghanistan were virtually nonexistent. The provinces in each conflict severity group were also different in the 2 study periods, given that conflict was dynamic and varied across time. Notwithstanding these limitations, we believe that because the main objective of this study was to assess associations with conflict severity, staying true to conflict criteria was more important than retaining consistent provinces.

## Conclusions

Afghanistan has made progress despite ongoing insecurity and conflict, yet several essential health interventions and the health system remain at risk of falling behind. Immediate attention and targeting appear to be needed to maintain and scale up gains toward achieving the United Nations’ Sustainable Development Goals. This will undoubtedly necessitate effective and strategic collaboration from international bodies, funders, government, NGOs, civilians, and local leaders.

## Notes

Supplement. **eMethods 1.** Evolution of NGO Contracting Models for Health Service Delivery in Afghanistan **eMethods 2.** Overview of Conflict Classification and Stepwise Modeling Approach **eTable 1.** Survey Sample Sizes **eTable 2.** Overview of Data Sources Used for Each of Our Analyses **eTable 3.** Reproductive, Maternal, Newborn and Child Health (RMNCH) Intervention Definitions **eTable 4.** Health Systems Performance Domains and Indicators **eTable 5.** Battle-Related Deaths (BRDs) by Province, 2003-2017 **eTable 6.** Crude and Multivariable Analysis of Conflict Association With RMNCH Coverage **eTable 7.** RMNCH Interventions Coverage by Conflict Status **eTable 8.** Adjusted Percentage Point Change in Key RMNCH Interventions by Conflict Status During 2003-2018 and 2010-2018 **eTable 9.** Health Systems Composite Indicators by Conflict Status **eTable 10.** Crude and Multivariable Analysis of Conflict Association With Health Systems Indicators **eFigure 1.** Average Annual Percentage Point Change in Composite Coverage Index vs Conflict by Province **eFigure 2.** Crude and Multivariable Analysis of Conflict Association With RMNCH Coverage **eFigure 3.** Health System Overall Mean Scores and Change by Province **eFigure 4.** Average Annual Percentage Point Change in Overall Health Systems Performance vs Conflict by Province **eFigure 5.** Crude and Multivariable Analysis of Conflict Association With Health Systems Indicators **eReferences.**
