# Supplementary material for: Association of Exposure to Civil Conflict With Maternal Resilience and Maternal and Child Health and Health System Performance in Afghanistan
Source: JAMA Netw Open. 2019 Nov 8;2(11):e1914819. doi: 10.1001/jamanetworkopen.2019.14819 (PMC6902774; doi:10.1001/jamanetworkopen.2019.14819)
Supplement: Supplement. — eMethods 1. Evolution of NGO Contracting Models for Health Service Delivery in Afghanistan eMethods 2. Overview of Conflict Classification and Stepwise Modeling Approach eTable 1. Survey Sample Sizes eTable 2. Overview of Data Sources Used for Each of Our Analyses eTable 3. Reproductive, Maternal, Newborn and Child Health (RMNCH) Intervention Definitions eTable 4. Health Systems Performance Domains and Indicators eTable 5. Battle-Related Deaths (BRDs) by Province, 2003-2017 eTable 6. Crude and Multivariable Analysis of Conflict Association With RMNCH Coverage eTable 7. RMNCH Interventions Coverage by Conflict Status eTable 8. Adjusted Percentage Point Change in Key RMNCH Interventions by Conflict Status During 2003-2018 and 2010-2018 eTable 9. Health Systems Composite Indicators by Conflict Status eTable 10. Crude and Multivariable Analysis of Conflict Association With Health Systems Indicators eFigure 1. Average Annual Percentage Point Change in Composite Coverage Index vs Conflict by Province eFigure 2. Crude and Multivariable Analysis of Conflict Association With RMNCH Coverage eFigure 3. Health System Overall Mean Scores and Change by Province eFigure 4. Average Annual Percentage Point Change in Overall Health Systems Performance vs Conflict by Province eFigure 5. Crude and Multivariable Analysis of Conflict Association With Health Systems Indicators eReferences. [file jamanetwopen-2-e1914819-s001.pdf]

Supplementary Online Content

Akseer N, Rizvi A, Bhatti Z, et al. Association of exposure to civil conflict with maternal resilience and maternal and child health and health system performance in Afghanistan. *JAMA Netw Open*. 2019;2(11):e1914819. doi:10.1001/jamanetworkopen.2019.14819

eMethods 1. Evolution of NGO Contracting Models for Health Service Delivery in Afghanistan
eMethods 2. Overview of Conflict Classification and Stepwise Modeling Approach
eTable 1. Survey Sample Sizes
eTable 2. Overview of Data Sources Used for Each of Our Analyses
eTable 3. Reproductive, Maternal, Newborn and Child Health (RMNCH) Intervention Definitions
eTable 4. Health Systems Performance Domains and Indicators
eTable 5. Battle-Related Deaths (BRDs) by Province, 2003-2017
eTable 6. Crude and Multivariable Analysis of Conflict Association With RMNCH Coverage
eTable 7. RMNCH Interventions Coverage by Conflict Status
eTable 8. Adjusted Percentage Point Change in Key RMNCH Interventions by Conflict Status During 2003-2018 and 2010-2018
eTable 9. Health Systems Composite Indicators by Conflict Status
eTable 10. Crude and Multivariable Analysis of Conflict Association With Health Systems Indicators
eFigure 1. Average Annual Percentage Point Change in Composite Coverage Index vs Conflict by Province
eFigure 2. Crude and Multivariable Analysis of Conflict Association With RMNCH Coverage
eFigure 3. Health System Overall Mean Scores and Change by Province
eFigure 4. Average Annual Percentage Point Change in Overall Health Systems Performance vs Conflict by Province
eFigure 5. Crude and Multivariable Analysis of Conflict Association With Health Systems Indicators
eReferences.

This supplementary material has been provided by the authors to give readers additional information about their work.

eMethods 1. Evolution of NGO Contracting Models for Health Service Delivery in Afghanistan

History and Rationale for Introducing Contracting Mechanisms

After decades of war, Afghanistan had virtually no health system and infrastructure. An effective and rapid approach was needed to scale up service delivery throughout the country, and specifically hard to reach areas. NGOs had a strong presence throughout the nation, and were keen and equipped with capacity, infrastructure and personnel to fill the gap. In consultation with development partners and key funders, the Afghan Ministry of Public Health (MoPH) adapted an NGO contracting mechanism to rapidly deliver the basic package of health services (BPHS) nationwide [10-12].

Evolution of the Contracting Models: Contracting In vs Contracting Out

Since 2003, the MOPH contracted well-established NGOs to deliver the services BPHS in a defined geographic area, which in most cases was a province. This process of "contracting-out" service delivery contracts to NGOs was implemented in 31 of 34 provinces. The MoPH was involved in selecting the NGOs through a competitive bid process, managing the contracts, and monitoring and evaluating performance. The contracts ranged from 12 to 36 months and NGOs were paid according to budgets that they submitted as part of their bids; full payment depended on achievement of agreed-on goals. Early on, NGOs were largely international, though over time, an increasing majority of contracts were awarded to Afghan NGOs. In 3 provinces (Parwan, Panjsher, Kapisa), the MoPH approach for service delivery was "contracting in" or recruiting managers to strengthen the existing MoPH. Coined the strengthening mechanism, this approach was designed to build capacity and strengthen service delivery in government facilities [10, 11].

The Essential Package of Hospital Services and Benefits Package

The Essential Package of Hospital Services (EPHS) was implemented in 2005 and was designed to complement the BPHS by specifying the in-patient clinical and ancillary services and defining staffing, equipment and drugs for the three types of hospitals: district, provincial and national. The link between the BPHS and EPHS health services is the district hospital, which serves as the first referral-level hospital for primary care facilities. With the two packages, the MOPH specified all the services, staffing and equipment expected at every level of the Afghan health system [10].

eMethods 2. Overview of Conflict Classification and Stepwise Modeling Approach

Conflict classification

Consultations with country experts confirmed that the classifications using only BRDs most accurately reflected province-level conflict severity. We explored BRD classifications based on death counts and rates (total deaths/100,000 population) for 3 and 5 year periods. For sensitivity analyses, the province conflict ranking and all conflict-stratified analyses were compared using each categorization. Though some variation was noted in the provinces' conflict categorization, using count- vs rate-based conflict classification did not substantially change our effect estimates or inferences. We thus proceeded with classification based on counts since it's the recommended approach by Uppsala and conflict severity were most closely aligned with realities on the ground.

Step-wise multivariable linear regression approach

To examine the association between conflict and child nutrition outcomes, we conducted a series of step-wise multivariable linear regression models using district-level (n=399) data. District-level child undernutrition outcomes (wasting, stunting, underweight, stunting & wasting) were obtained from Akseer et al 2018's Bayesian geospatial analysis of NNS 2013 data [1]. Conflict was measured using 4-year battle-related death totals for each district with a log transformation to account for right skewness. The 4-year lag period (2010-2013) was selected to align NNS 2013 anthropometry data with an appropriate conflict exposure period and potential impact on child growth and adverse nutrition outcomes [2]. Analyses were conducted in 3 stages (models) using ordinary least squares (OLS) regression, adjusting for potential sources of confounding related to nutritional outcomes [3]. Model 1 was simple crude associations between the exposure and outcome variables. Model 2 included model 1 + adjustments for household poverty (% population in the poorest 2 quintiles) and maternal illiteracy. Model 3 included model 2 + adjustments for household lack of access to improved water and sanitation sources. At each step, the crude and adjusted association between the conflict exposure and outcome was analyzed for statistical significance. Multicollinearity among adjustment variables was evaluated using variance inflation factors (VIF) where VIF>3 were considered suspect for collinearity. Model diagnostics such as graphical conditional studentized residuals and fixed effect influence statistics were accessed to assess model fit. Model parameters (β) were estimated with 95% confidence intervals and standardized βs were estimated to determine relative ranking (importance) of model covariables to each outcomes. Statistical significance was established at $p<0.05$ and SAS v 9.4 was used for analysis.

eTable 1. Survey Sample Sizes

Survey	Under-5 Sample Size	Women age 15-49 years Sample Size
MICS 2003/04	25,777	21,677
MICS 2010/11	15,320	22,052
NNS 2013	24,348	26,901
AHS 2018	23,133	21,096*

Abbreviations: AHS: Afghanistan Health Survey, MICS: Multiple Indicator Cluster Survey, NNS: National Nutrition Survey.

*women were defined as 12-49 years

eTable 2. Overview of Data Sources Used for Each of Our Analyses

Outcome(s)	Data Source(s)	Analytical Strategy
Child anthropometry	Afghanistan National Nutrition Survey (NNS) 2013 / Akseer et al. (2018)	Hierarchical multivariable linear regression models using district-level (n=399) data
RMNCH	Multiple Indicator Cluster Surveys (MICS) 2004, MICS 2010-11, Afghanistan Health Survey (AHS) 2018	Difference-in-difference (DID) analysis using generalized linear models and generalized estimating equations
Health system performance	Afghanistan Balanced Scorecards (BSC) 2004-2016	Difference-in-difference (DID) analysis using generalized linear models and generalized estimating equations

eTable 3. Reproductive, Maternal, Newborn and Child Health (RMNCH) Intervention Definitions

Indicator	Definition
Current use of contraceptives (any method) (CPU)	Prevalence of current contraceptive use among married women 15-49 years old, any method
Current use of contraceptives (any modern method) (CPUM)	Prevalence of current contraceptive use among married women 15-49 years old, any modern method
At least one antenatal care (ANC) checkup from a skilled provider	Percentage of women attended at least once during pregnancy by skilled health personnel
Skilled birth attendance (SBA) at last delivery	Percentage of live births attended by skilled health personnel
Facility births	Percentage of births delivered in a health facility
BCG vaccination (BCG)	The percentage of children aged 12-23 months who have received at least one dose of Bacillus Calmette-Guérin vaccine

© 2019 Akseer N et al. *JAMA Network Open*

DPT3/Penta 3 vaccination (DPT3)	The percentage of one-year-olds who have received three doses of the combined diphtheria, tetanus toxoid and pertussis (DTP3) vaccine in a given year
Measles vaccination (MSL)	The percentage of children aged 12-23 months who have received at least one dose of measles-containing vaccine in a given year
Care seeking for Acute Respiratory Infection (ARI)	Percentage of children under age five with symptoms of acute respiratory illness (ARI) during the two-week period before the survey and, among children with symptoms of ARI, the percentage who were taken to an appropriate health provider
Oral Rehydration Therapy (ORT) use for diarrhea	Percentage of children under age five ill with diarrhea during the two-week period before the survey and, among children ill with diarrhea, the percentage who received Oral Rehydration Solution (ORS) (ORS packet or pre-packaged ORS fluid)
Composite Coverage Index (CCI)	The CCI is a weighted average of 8 core indicators: demand for family planning satisfied, ANC 1+ visit, skilled birth attendance, BCG vaccine, 3 doses of DPT vaccine, measles vaccination, oral rehydration with continued feeding for diarrhea treatment, and care seeking for acute respiratory infection<br>$CCI = \frac{1}{4}\left(FPS + \frac{SBA + ANC}{2} + \frac{2\,DPT3 + MSL + BCG}{4} + \frac{ORT + ARI}{2}\right)$

© 2019 Akseer N et al. *JAMA Network Open*

eTable 4. Health Systems Performance Domains and Indicators

Note: All domains and indicators measured as percentages ranging from 0-100 where higher values indicate better performance.

Domain	Definition
Domain A: Client and community feedback	A composite score examining viewpoints of clients of BPHS facilities and community members on overall client satisfaction, perceived quality of care and community involvement in decision-making.
Domain B: Human resource	A composite score documenting experience of BPHS facility employees on health worker satisfaction, motivation, salary, staffing, provider knowledge and staff training.
Domain C: Physical capacity	A composite score measuring the BPHS facility's capacity to provide health care in the following areas: equipment functionality, pharmaceuticals and vaccine availability, laboratory functionality, existence of clinical guidelines, and physical infrastructure (e.g. heat, water, etc).
Domain D: Quality of service provision	A composite score measuring the technical aspects of service delivery and the patient-provider interaction through examining appropriateness of: client background and physical assessment, patient counseling, precautions and safety measures to prevent infection and injury, and time spent with the patient.
Domain E: Management systems	A composite score that measures functions related to the management of health facilities including use of health management information systems (HMIS), financial systems, and facility management functionality.
Domain F: Overall mission	A composite score that measures the extent to which the facility promotes greater access to health care for females and the poor.
Overall means	An overall ranking of health systems performance based on above domains.

© 2019 Akseer N et al. *JAMA Network Open*

Select component indicators	• Equipment functionality index<br>• Drug availability index (post-2010: Pharmaceuticals and vaccines availability index)<br>• Infrastructure index (post-2010: Functional infrastructure index)<br>• Patient history and physical examination index (post-2010: Client background and physical assessment index)<br>• Patient counseling index (post-2010: Client counseling index)<br>• Female health worker index<br>• Provider knowledge score

© 2019 Akseer N et al. *JAMA Network Open*

eTable 5. Battle-Related Deaths (BRDs) by Province, 2003-2017

Province	2003	2004	2005	2006	2007	2008	2009	2010	2011	2012	2013	2014	2015	2016	2017
Badakhshan	0	0	0	0	12	0	5	19	32	131	338	234	561	375	383
Badghis	57	5	0	8	124	117	242	257	140	129	150	58	157	209	316
Baghlan	0	0	0	1	12	11	106	411	128	111	175	114	291	636	375
Balkh	84	7	0	4	1	0	16	33	89	76	53	131	61	199	285
Bamyan	0	0	0	0	0	0	0	1	4	22	0	0	0	1	7
Daykundi	0	0	18	2	0	0	0	49	33	1	11	9	2	1	2
Farah	0	13	9	64	343	245	426	136	174	359	340	247	391	621	633
Faryab	26	0	1	0	0	11	27	91	184	245	250	244	454	499	894
Ghazni	24	15	39	193	426	393	277	294	357	369	588	805	707	1287	913
Ghor	0	0	0	2	0	12	39	22	11	80	33	113	221	188	166
Hilmand	40	84	248	1316	2123	1320	1205	1316	1024	1097	1038	1272	2516	2961	2250
Hirat	1	42	4	74	169	133	189	151	210	167	298	370	417	498	451
Jawzjan	3	0	0	0	0	1	26	26	115	42	107	78	171	370	497
Kabul	6	8	14	74	142	160	145	133	193	242	169	239	239	431	448
Kandahar	215	175	378	1444	1197	843	680	769	606	641	525	550	787	950	1330
Kapisa	0	0	0	13	67	110	82	71	127	128	125	55	180	76	236
Khost	41	50	58	118	89	291	342	298	206	240	78	205	214	194	162
Kunar	4	39	103	138	178	156	211	501	730	361	348	639	317	290	217
Kunduz	0	0	0	0	35	16	453	507	199	199	259	464	1755	1541	1217
Laghman	0	1	29	24	17	42	98	68	117	188	151	221	179	136	414
Logar	11	0	12	4	29	34	134	88	206	301	368	323	349	211	364
Nangarhar	4	9	6	28	53	123	97	260	367	383	572	749	1514	2980	2872
Nimroz	5	15	2	44	41	176	108	163	118	128	67	92	97	71	88
Nuristan	1	3	12	62	92	36	235	130	292	124	84	91	108	41	222
Paktika	75	10	112	255	382	252	251	302	448	229	153	261	374	414	516
Paktya	8	3	17	52	131	160	136	219	197	212	233	219	143	494	443
Panjsher	0	0	0	0	0	0	1	0	7	0	11	0	0	0	0
Parwan	0	0	0	3	24	10	11	19	79	40	75	91	58	74	93
Samangan	25	0	0	0	0	0	3	0	2	33	3	11	34	35	19
Sari Pul	79	0	0	0	0	0	0	11	64	65	70	137	226	306	340
Takhar	0	0	4	0	0	1	13	110	55	18	8	68	213	211	243
Uruzgan	93	118	286	740	791	473	397	257	186	233	266	207	592	1056	1239
Wardak	0	0	2	16	79	119	131	147	306	298	251	326	224	154	269

© 2019 Akseer N et al. *JAMA Network Open*

Province	2003	2004	2005	2006	2007	2008	2009	2010	2011	2012	2013	2014	2015	2016	2017
**Zabul**	140	219	345	297	437	398	361	235	189	168	184	257	459	203	407

© 2019 Akseer N et al. *JAMA Network Open*

eTable 6. Crude and Multivariable Analysis of Conflict Association With RMNCH Coverage

A, 2003-2010

Skilled birth attendance	**Bivariate**			**Main Effect model w/o covariates**			**Adjusted**		
	Coef.	***95% CI***	***p-value***	***Coef.***	***95% CI***	***p-value***	***Coef.***	***95% CI***	***p-value***
**Conflict Type**									
0=Minimal	ref.						ref.		
1=Moderate	0.57	(0.37,0.77)	<.0001	-1.27	(-1.82,-0.71)	<.0001	-0.89	(-1.35, -0.43)	<.0001
2=Severe	-0.21	(-0.63,0.20)	0.314	-1.21	(-2.32,-0.11)	0.031	-1.35	(-2.54, -0.16)	0.026
**Year**	1.32	(1.09,1.55)	<.0001	1.01	(0.74,1.28)	<.0001	1.32	(1.08, 1.56)	<.0001
**Conflict Type 1*time**	--			0.84	(0.47,1.21)	<.0001	0.53	(0.20, 0.86)	0.002
**Conflict Type 2*time**	--			0.41	(-0.33,1.14)	0.278	0.43	(-0.38, 1.24)	0.294
% Women illiterate	-1.93	(-2.14, -1.72)	<.0001				-1.18	(-1.37, -0.98)	<.0001
Contracting type	0.34	(-0.03,0.71)	0.069				-0.11	(-0.44, 0.23)	0.537
Rural population	-2.00	(-2.22,-1.79)	<.0001				-2.00	(-2.23, -1.78)	0.150

Measles vaccination	**Bivariate**			**Main Effect model w/o covariates**			**Adjusted**		
	Coef.	***95% CI***	***p-value***	***Coef.***	***95% CI***	***p-value***	***Coef.***	***95% CI***	***p-value***
**Conflict Type**									
0=Minimal	ref.						ref.		
1=Moderate	-0.17	(-0.45, 0.10)	0.213	-0.46	(-1.09, 0.18)	0.156	-0.73	(-1.40, -0.06)	0.034
2=Severe	0.15	(-0.33, 0.64)	0.540	1.24	(0.03, 2.44)	0.044	1.33	(0.13, 2.53)	0.031
**Year**	0.73	(0.54, 0.92)	<.0001	0.71	(0.42, 1.00)	<.0001	0.70	(0.40, 1.00)	<.0001
**Conflict Type 1*time**	--			0.28	(-0.14, 0.70)	0.196	0.47	(0.00, 0.94)	0.044

© 2019 Akseer N et al. *JAMA Network Open*

**Conflict Type 2*time**	--			-1.08	(-1.95, -0.21)	0.015	-1.15	(-2.03, -0.27)	0.010
% Women illiterate	1.72	(1.14, 2.30)	<.0001				1.39	(0.79, 1.99)	<.0001
Contracting type	-0.18	(-0.77, 0.42)	0.561				-0.02	(-0.63, 0.59)	0.944
Rural population	0.95	(0.59, 1.30)	<.0001				0.76	(0.38, 1.15)	<.0001
Rural population	-0.19	(-0.62, 0.23)	0.370				-0.19	(-0.68, 0.30)	0.003

	**Bivariate**			**Main Effect model w/o covariates**			**Adjusted**		
Care-seeking for ARI	***Coef.***	***95% CI***	***p-value***	***Coef.***	***95% CI***	***p-value***	***Coef.***	***95% CI***	***p-value***
**Conflict Type**									
0=Minimal	ref.						ref.		
1=Moderate	-0.24	(-0.57, 0.08)	0.135	-0.91	(-1.60 ,-0.21)	0.011	-0.88	(-1.58 , -0.17)	0.015
2=Severe	0.32	(-0.20, 0.84)	0.222	0.49	(-0.83 , 1.80)	0.469	0.56	(-0.75 , 1.88)	0.401
**Year**	0.58	(0.38, 0.78)	<.0001	0.32	(0.07 , 0.56)	0.013	0.38	(0.11 , 0.65)	0.006
**Conflict Type 1*time**	--			0.65	(0.23 , 1.07)	0.002	0.59	(0.16 , 1.02)	0.008
**Conflict Type 2*time**	--			-0.16	(-1.13, 0.80)	0.739	-0.30	(-1.29 , 0.69)	0.555
% Women illiterate	0.21	(-0.18 , 0.59)	0.291				0.34	(-0.11 , 0.8)	0.137
Contracting type	0.45	(-0.04 , 0.94)	0.073				0.50	(0.00 , 1.00)	0.051
Rural population	-0.19	(-0.62 , 0.23)	0.370				-0.19	(-0.68 , 0.3)	0.003

	**Bivariate**			**Main Effect model w/o covariates**			**Adjusted**		
BCG	***Coef.***	***95% CI***	***p-value***	***Coef.***	***95% CI***	***p-value***	***Coef.***	***95% CI***	***p-value***
**Conflict Type**									
0=Minimal	ref.						ref.		
1=Moderate	0.42	(0.17, 0.66)	0.001	0.65	(0.05, 1.24)	0.033	0.35	(-0.31, 1.00)	0.296
2=Severe	0.12	(-0.40, 0.63)	0.652	1.36	(0.18, 2.55)	0.024	1.52	(0.32, 2.73)	0.014
**Year**	1.03	(0.84, 1.23)	<.0001	1.15	(0.89, 1.41)	<.0001	1.15	(0.86, 1.44)	<.0001
**Conflict Type 1*time**	--			-0.24	(-0.65, 0.18)	0.263	0.05	(-0.45, 0.55)	0.851

© 2019 Akseer N et al. *JAMA Network Open*

**Conflict Type 2*time**	--			-1.24	(-2.05, -0.43)	0.003	-1.33	(-2.17, -0.49)	0.002
% Women illiterate	1.62	(1.15, 2.08)	<.0001				1.02	(0.53, 1.51)	<.0001
Contracting type	-0.45	(-0.90, -0.01)	0.047				-0.41	(-0.84, 0.01)	0.055
Rural population	1.39	(1.16, 1.62)	<.0001				1.22	(0.99, 1.45)	<.0001

	**Bivariate**			**Main Effect model w/o covariates**			**Adjusted**		
DPT3/Penta	***Coef.***	***95% CI***	***p-value***	***Coef.***	***95% CI***	***p-value***	***Coef.***	***95% CI***	***p-value***
**Conflict Type**									
0=Minimal	ref.						ref.		
1=Moderate	-0.32	(-0.55, -0.08)	0.008	-0.57	(-1.16, 0.02)	0.060	-0.25	(-0.85, 0.35)	0.409
2=Severe	-0.32	(-0.83, 0.20)	0.232	1.67	(0.44, 2.90)	0.008	1.66	(0.33, 2.98)	0.014
**Year**	0.16	(-0.04, 0.35)	0.108	0.23	(-0.06, 0.52)	0.116	0.41	(0.09, 0.72)	0.013
**Conflict Type 1*time**	--			0.24	(-0.18, 0.67)	0.256	-0.07	(-0.51, 0.38)	0.769
**Conflict Type 2*time**	--			-1.97	(-2.85, -1.10)	<.0001	-2.11	(-3.05, -1.17)	<.0001
% Women illiterate	-1.31	(-1.74, -0.88)	<.0001				-0.49	(-0.98, -0.01)	0.046
Contracting type	0.80	(0.22, 1.37)	0.007				0.54	(-0.04, 1.12)	0.068
Rural population	-1.65	(-1.92, -1.37)	<.0001				-1.52	(-1.81, -1.23)	0.786

	**Bivariate**			**Main Effect model w/o covariates**			**Adjusted**		
ORT	***Coef.***	***95% CI***	***p-value***	***Coef.***	***95% CI***	***p-value***	***Coef.***	***95% CI***	***p-value***
**Conflict Type**									
0=Minimal	ref.						ref.		
1=Moderate	0.45	(0.13, 0.78)	0.006	0.66	(-0.08 ,1.41)	0.081	1.06	(0.3, 1.82)	0.006
2=Severe	-1.61	(-2.14, -1.08)	<.0001	-4.47	(-5.63 ,-3.31)	<.0001	-4.39	(-5.48, -3.31)	<.0001
**Year**	1.00	(0.75, 1.24)	<.0001	0.86	(0.6 ,1.12)	<.0001	1.02	(0.73, 1.31)	<.0001
**Conflict Type 1*time**	--			-0.21	(-0.7 ,0.28)	0.396	-0.43	(-0.97, 0.1)	0.111
**Conflict Type 2*time**	--			2.81	(2.08 ,3.53)	<.0001	2.78	(2.06, 3.5)	<.0001
% Women illiterate	-0.96	(-1.27, -0.64)	<.0001				-0.46	(-0.89, -0.02)	0.039
Contracting type	0.13	(-0.29, 0.54)	0.550				-0.28	(-0.67, 0.11)	0.163
Rural population	-1.26	(-1.6, -0.93)	<.0001				-1.30	(-1.65, -0.94)	0.454

	**Bivariate**			**Main Effect model w/o covariates**			**Adjusted**		
Contraceptive prevalence (any method)	***Coef.***	***95% CI***	***p-value***	***Coef.***	***95% CI***	***p-value***	***Coef.***	***95% CI***	***p-value***

© 2019 Akseer N et al. *JAMA Network Open*

**Conflict Type**									
0=Minimal	ref.						ref.		
1=Moderate	0.49	(0.35, 0.62)	<.0001	-1.63	(-2.17, -1.09)	<.0001	-1.52	(-2.03, -1.01)	<.0001
2=Severe	0.79	(0.6, 0.99)	<.0001	-1.51	(-2.34, -0.67)	<.0001	-1.52	(-2.35, -0.7)	<.0001
**Year**	0.63	(0.5, 0.76)	<.0001	0.08	(-0.1, 0.27)	0.380	0.17	(0, 0.35)	0.050
**Conflict Type 1*time**	--			1.14	(0.83, 1.45)	<.0001	1.03	(0.73, 1.32)	<.0001
**Conflict Type 2*time**	--			1.21	(0.73, 1.69)	<.0001	1.22	(0.74, 1.69)	<.0001
% Women illiterate	-0.24	(-0.44, -0.04)	0.018				0.38	(0.22, 0.53)	<.0001
Contracting type	0.04	(-0.21, 0.3)	0.741				-0.42	(-0.7, -0.14)	0.003
Rural population	-1.08	(-1.26, -0.91)	<.0001				-1.16	(-1.32, -1.01)	<.0001

ANC	**Bivariate**			**Main Effect model w/o covariates**			**Adjusted**		
	Coef.	***95% CI***	***p-value***	***Coef.***	***95% CI***	***p-value***	***Coef.***	***95% CI***	***p-value***
**Conflict Type**									
0=Minimal	ref.						ref.		
1=Moderate	0.60	(0.42, 0.79)	<.0001	-1.10	(-1.74 , -0.46)	0.001	-0.75	(-1.33, -0.17)	0.012
2=Severe	-0.06	(-0.41, 0.28)	0.730	-1.48	(-2.68 , -0.28)	0.016	-1.70	(-2.88, -0.51)	0.005
**Year**	1.60	(1.4, 1.81)	<.0001	1.35	(1.09 , 1.61)	<.0001	1.69	(1.45, 1.93)	<.0001
**Conflict Type 1*time**	--			0.71	(0.31 , 1.1)	<.0001	0.43	(0.06, 0.8)	0.024
**Conflict Type 2*time**	--			0.57	(-0.13 , 1.28)	0.111	0.66	(-0.05, 1.37)	0.070
% Women illiterate	-1.82	(-2.02, -1.62)	<.0001				-1.07	(-1.28, -0.86)	<.0001
Contracting type	0.19	(-0.17, 0.54)	0.299				-0.27	(-0.56, 0.03)	0.081
Rural population	-1.89	(-2.1, -1.67)	<.0001				-2.02	(-2.27, -1.77)	0.037

B, 2010-2018

Skilled birth attendance	**Bivariate**			**Main Effect model w/o covariates**			**Adjusted**		
	Coef.	***95% CI***	***p-value***	***Coef.***	***95% CI***	***p-value***	***Coef.***	***95% CI***	***p-value***
**Conflict Type**									
0=Minimal	ref.						ref.		

© 2019 Akseer N et al. *JAMA Network Open*

1=Moderate	0.41	(0.12, 0.69)	0.005	1.15	(0.52 , 1.79)	<.0001	0.79	(0.18, 1.39)	0.011
2=Severe	-0.36	(-0.89, 0.16)	0.173	-0.41	(-1.55, 0.73)	0.479	-0.49	(-1.65, 0.67)	0.410
**Year**	0.83	(0.64, 1.02)	<.0001	1.33	(1.06, 1.61)	<.0001	1.13	(0.88, 1.39)	<.0001
**Conflict Type 1*time**	--			-0.75	(-1.15, -0.35)	<.0001	-0.53	(-0.89, -0.16)	0.005
**Conflict Type 2*time**	--			0.09	(-0.63 , 0.64)	0.980	0.10	(-0.55, 0.75)	0.764
% Women illiterate	-1.65	(-1.90 ,-1.40)	<.0001				-1.17	(-1.52, -0.92)	<.0001
Contracting type	-0.25	(-0.63, -0.13)	0.198				-0.55	(-0.95, -0.16)	0.006
Rural population	-1.89	(-2.17, -1.62)	<.0001				-1.66	(-1.95, -1.38)	<.0001

Measles vaccination	**Bivariate**			**Main Effect model w/o covariates**			**Adjusted**		
	Coef.	***95% CI***	***p-value***	***Coef.***	***95% CI***	***p-value***	***Coef.***	***95% CI***	***p-value***
**Conflict Type**									
0=Minimal	ref.						ref.		
1=Moderate	0.08	(-0.14, 0.30)	0.471	0.50	(-0.22, 1.22)	0.176	0.32	(-0.42, 1.06)	0.393
2=Severe	-0.24	(-0.58, 0.11)	0.183	-1.22	(-2.73, 0.29)	0.115	-1.24	(-2.78, 0.30)	0.113
**Year**	1.20	(0.99, 1.40)	<.0001	1.49	(1.16, 1.83)	<.0001	1.39	(1.05, 1.73)	0.186
**Conflict Type 1*time**				-0.40	(-0.85, 0.04)	0.076	-0.30	(-0.74, 0.14)	0.440
**Conflict Type 2*time**				0.29	(-0.54, 1.12)	0.494	0.33	(-0.51, 1.17)	<.0001
% Women illiterate	-0.96	(-1.18, -0.73)	<.0001				-0.68	(-0.91, -0.45)	0.889
Contracting type	0.01	(-0.40, 0.41)	0.971				-0.03	(-0.46, 0.40)	<.0001
Rural population	1.12	(-0.96, -0.46)	<.0001				-0.53	(-0.77, -0.29)	0.076

Care-seeking for ARI	**Bivariate**			**Main Effect model w/o covariates**			**Adjusted**		
	Coef.	***95% CI***	***p-value***	***Coef.***	***95% CI***	***p-value***	***Coef.***	***95% CI***	***p-value***
**Conflict Type**									
0=Minimal	ref.						ref.		
1=Moderate	0.44	(0.21, 0.66)	<.0001	0.48	(-0.21, 1.17)	0.175	0.49	(-0.22, 1.19)	0.177

© 2019 Akseer N et al. *JAMA Network Open*

2=Severe	0.43	(-0.05, 0.92)	0.081	0.06	(-1.67, 1.78)	0.949	0.07	(-1.69, 1.82)	0.940
**Year**	0.40	(0.14, 0.67)	0.003	0.38	(0.06, 0.71)	0.020	0.32	(-0.01, 0.64)	0.054
**Conflict Type 1*time**				-0.08	(-0.57, 0.41)	0.752	-0.03	(-0.51, 0.45)	0.908
**Conflict Type 2*time**				0.11	(-0.90, 1.11)	0.842	0.12	(-0.88, 1.12)	0.810
% Women illiterate	-0.38	(-0.73, -0.03)	0.031				-0.32	(-0.65, 0.01)	0.057
Contracting type	-0.01	(-0.29, 0.26)	0.927				-0.37	(-0.65, 0.01)	0.017
Rural population	-0.13	(-0.49, 0.23)	0.469				0.07	(-0.27, 0.41)	0.696

BCG	**Bivariate**			**Main Effect model w/o covariates**			**Adjusted**		
	Coef.	***95% CI***	***p-value***	***Coef.***	***95% CI***	***p-value***	***Coef.***	***95% CI***	***p-value***
**Conflict Type**									
0=Minimal	ref.						ref.		
1=Moderate	-0.39	(-0.61, -0.16)	0.001	1.59	(0.82, 2.36)	<.0001	1.39	(0.63, 2.16)	<.0001
2=Severe	-0.37	(-0.74, -0.01)	0.043	-1.15	(-2.55, 0.25)	0.106	-1.20	(-2.63, 0.23)	0.099
**Year**	0.64	(0.43, 0.85)	<.0001	1.69	(1.30, 2.08)	<.0001	1.54	(1.15, 1.93)	<.0001
**Conflict Type 1*time**				-1.42	(-1.91, -0.92)	<.0001	-1.30	(-1.79, -0.81)	<.0001
**Conflict Type 2*time**				0.03	(0.78, 0.84)	0.939	0.06	(-0.75. 0.88)	0.879
% Women illiterate	-1.15	(-1.42, -0.88)	<.0001				-0.84	(-1.11, -0.57)	<.0001
Contracting type	-0.31	(-0.74, 0.13)	0.168				0.10	(-0.34, 0.55)	0.643
Rural population	-0.71	(-0.98, -0.43)	<.0001				-0.52	(-0.80. -0.24)	<.0001

DPT3/Penta	**Bivariate**			**Main Effect model w/o covariates**			**Adjusted**		
	Coef.	***95% CI***	***p-value***	***Coef.***	***95% CI***	***p-value***	***Coef.***	***95% CI***	***p-value***
**Conflict Type**									
0=Minimal	ref.						ref.		
1=Moderate	-0.42	(-0.64, -0.20)	<.0001	0.94	(0.18, 1.70)	0.016	0.68	(-0.08, 1.44)	0.078
2=Severe	-0.13	(-0.51, 0.25)	0.510	-3.82	(-5.4, -2.23)	<.0001	-3.85	(-5.41, -2.30)	<.0001
**Year**	1.15	(0.93, 1.37)	<.0001	1.85	(1.50, 2.21)	<.0001	1.71	(1.35, 2.07)	<.0001

© 2019 Akseer N et al. *JAMA Network Open*

**Conflict Type 1*time**				-1.02	(-1.47, 0.56)	<.0001	-0.88	(-1.33, -0.43)	<.0001
**Conflict Type 2*time**				1.54	(0.69, 2.39)	<.0001	1.56	(0.71, 2.41)	<.0001
% Women illiterate	-1.18	(-1.40, -0.96)	<.0001				-0.90	(-1.13, -0.67)	<.0001
Contracting type	-0.16	(-0.53, 0.21)	0.404				0.24	(-0.09, 0.56)	0.151
Rural population	-0.60	(-0.83, -0.36)	<.0001				-0.40	(-0.64, -0.15)	0.002

ORT	**Bivariate**			**Main Effect model w/o covariates**			**Adjusted**		
	Coef.	***95% CI***	***p-value***	***Coef.***	***95% CI***	***p-value***	***Coef.***	***95% CI***	***p-value***
**Conflict Type**									
0=Minimal	ref.						ref.		
1=Moderate	0.17	(-0.07, 0.40)	0.161	0.16	(-0.63, 0.94)	0.695	0.23	(-0.55, 1.02)	0.556
2=Severe	0.13	(-0.21, 0.47)	0.448	2.09	(0.95, 3.24)	<.0001	2.09	(0.93, 3.25)	<.0001
**Year**	-0.53	(-0.76, -0.29)	<.0001	-0.56	(-0.85, -0.27)	<.0001	-0.49	(-0.78, -0.20)	0.001
**Conflict Type 1*time**				0.08	(-0.37, 0.53)	0.718	0.03	(-0.41, 0.48)	0.887
**Conflict Type 2*time**				-0.95	(-1.60, -0.29)	0.005	-0.97	(-1.63, -0.31)	0.004
% Women illiterate	0.40	(0.16, 0.65)	0.001				0.19	(-0.06, 0.44)	0.143
Contracting type	0.36	(0.02, 0.71)	0.039				0.29	(-0.09, 0.66)	0.131
Rural population	0.33	(0.11, 0.54)	0.004				0.28	(0.07, 0.50)	0.009

Contraceptive Prevalence (any method)	**Bivariate**			**Main Effect model w/o covariates**			**Adjusted**		
	Coef.	***95% CI***	***p-value***	***Coef.***	***95% CI***	***p-value***	***Coef.***	***95% CI***	***p-value***
**Conflict Type**									
0=Minimal	ref.						ref.		
1=Moderate	0.64	(0.46, 0.82)	<.0001	1.47	(1.05, 1.89)	<.0001	1.52	(1.09, 1.95)	<.0001
2=Severe	0.92	(0.66, 1.18)	<.0001	1.47	(0.86, 2.07)	<.0001	1.42	(0.82, 2.02)	<.0001
**Year**	0.69	(0.54, 0.84)	<.0001	1.14	(0.92, 1.37)	<.0001	1.13	(0.91, 1.34)	<.0001
**Conflict Type 1*time**				-0.83	(-1.11, -0.54)	<.0001	-0.85	(-1.14, -0.57)	<.0001
**Conflict Type 2*time**				-0.55	(-0.95, -0.14)	0.008	-0.50	(-0.92, -0.08)	0.020

© 2019 Akseer N et al. *JAMA Network Open*

% Women illiterate	0.37	(0.24, 0.50)	<.0001				0.68	(0.57, 0.80)	<.0001
Contracting type	-0.04	(-0.33, 0.24)	0.759				-0.69	(-1.03, -0.36)	<.0001
Rural population	-0.68	(-0.84, -0.53)	<.0001				-0.83	(-0.99, -0.67)	<.0001

ANC	Bivariate			Main Effect model w/o covariates			Adjusted		
	Coef.	*95% CI*	*p-value*	*Coef.*	*95% CI*	*p-value*	*Coef.*	*95% CI*	*p-value*
**Conflict Type**									
0=Minimal	ref.						ref.		
1=Moderate	0.30	(0.04, 0.57)	0.025	1.09	(0.53, 1.65)	<.0001	0.86	(0.30, 1.42)	0.003
2=Severe	-0.34	(-0.74, 0.05)	0.089	-0.31	(-1.18, 0.55)	0.478	-0.25	(-1.17, 0.66)	0.587
**Year**	-0.63	(-0.78, -0.47)	<.0001	-0.12	(-0.33, 0.08)	0.227	-0.47	(-0.71, -0.23)	<.0001
**Conflict Type 1*time**				-0.77	(-1.08, -0.46)	<.0001	-0.63	(-0.97, -0.30)	<.0001
**Conflict Type 2*time**				-0.02	(-0.50, 0.45)	0.921	-0.01	(-0.59, 0.57)	0.972
% Women illiterate	-1.56	(-1.81, -1.32)	<.0001				-1.18	(-1.44, -0.92)	<.0001
Contracting type	-0.49	(-0.82, -0.16)	0.004				-0.72	(-1.09, -0.35)	<.0001
Rural population	-1.50	(-1.75, -1.25)	<.0001				-1.28	(-1.53, -1.03)	<.0001

© 2019 Akseer N et al. *JAMA Network Open*

eTable 7. RMNCH Interventions Coverage by Conflict Status

A, 2003-2010

Indicator	Survey	N	Minimal Conflict	N	Moderate Conflict	N	Severe Conflict	Minimal vs. Moderate p-value	Minimal vs. Severe p-value
Contraceptives (Any method)	Mean % 2003	12,048	5.2	5,654	20.3	3,260	6.6	<.0001	0.013
	Mean % 2010	10,042	12.7	5,801	22.8	2,231	25.1	<.0001	<.0001
	Mean difference		7.5		2.5		18.5	<.0001	<.0001
Contraceptives (Any modern method)	Mean % 2003	12,033	4.4	5,647	16.2	3,255	6.3	<.0001	0.001
	Mean % 2010	10,042	11.8	5,801	20.1	2,231	24.6	<.0001	<.0001
	Mean difference		7.4		3.8		18.3	<.0001	<.0001
ANC by skilled provider	Mean % 2003	6,295	8.7	2,890	31.8	1,702	10.5	<.0001	0.330
	Mean % 2010	2,714	45.7	1,808	56.3	352	36.8	0.007	0.068
	Mean difference		37.0		24.5		26.3	<.0001	0.031
Facility deliveries	Mean % 2003	6,370	5.4	2,919	28.3	1,719	7.7	<.0001	0.011
	Mean % 2010	2,786	27.3	1,820	45.3	356	15.3	<.0001	0.013
	Mean difference		21.9		17.0		7.6	<.0001	0.001
SBA	Mean % 2003	6,380	7.4	2,915	29.5	1,720	8.3	<.0001	0.384
	Mean % 2010	2,786	33.4	1820	49.9	356	23.3	<.0001	0.037
	Mean difference		26.1		20.3		15.0	<.0001	0.021
BCG	Mean % 2003	2,505	41.6	1,283	19.2	857	49.3	<.0001	0.010
	Mean % 2010	1,429	60.9	816	71.8	247	34.8	0.026	<.0001
	Mean difference		19.3		52.5		-14.6	<.0001	<.0001
DPT3/Penta	Mean % 2003	2,504	23.3	1,283	45.5	857	11.7	<.0001	<.0001
	Mean % 2010	1,400	31.8	793	37.8	240	7.0	0.168	<.0001
	Mean difference		8.4		-7.7		-4.7	0.001	0.007
Measles	Mean % 2003	2,516	27.7	1,285	17.2	851	28.6	0.001	0.733
	Mean % 2010	1,413	39.9	817	47.7	244	20.4	0.076	0.003
	Mean difference		12.2		30.5		-8.2	<.0001	0.006
Care seeking for ARI	Mean % 2003	3,110	45.0	1,121	54.4	487	36.2	0.004	0.025
	Mean % 2010	1,696	57.0	1,041	65.7	212	61.8	0.020	0.546
	Mean difference		12.0		11.3		25.7	0.967	0.126
ORT	Mean % 2003	4,800	25.9	2,071	41.6	865	17.2	<.0001	0.007
	Mean % 2010	2,059	46.3	1,044	58.6	337	74.0	0.034	<.0001

© 2019 Akseer N et al. *JAMA Network Open*

	Mean difference		20.4		17.0		56.8	0.400	<.0001

B, 2010-2018

Indicator	Survey	N	Minimal Conflict	N	Moderate Conflict	N	Severe Conflict	Minimal vs. Moderate p-value	Minimal vs. Severe p-value
Contraceptives (Any method)	Mean % 2010	4,426	14.1	7,329	16.4	6,319	20.7	0.099	<.0001
	Mean % 2018	2,222	27.8	4,994	23.7	6,920	32.3	0.049	0.053
	Mean difference		13.7		7.3		11.6	0.011	0.075
Contraceptives (Any modern method)-	Mean % 2010	4,426	12.7	7,329	14.4	6,319	19.8	0.212	<.0001
	Mean % 2018	2,222	25.8	4,994	19.0	6,920	28.5	<.0001	0.208
	Mean difference		13.0		4.5		8.7	0.001	0.007
ANC by skilled provider	Mean % 2010	1,223	50.2	1,954	49.9	1,697	46.7	0.933	0.350
	Mean % 2018	3,099	42.1	7,440	34.2	10,367	32.6	<.0001	<.0001
	Mean difference		8.1		15.7		14.0	0.081	0.145
Facility deliveries	Mean % 2010	1,228	32.3	1,974	37.6	1,760	27.3	0.195	0.195
	Mean % 2018	1,402	62.9	3,395	46.7	4,529	59.4	0.005	0.008
	Mean difference		30.7		9.0		32.1	<.0001	0.718
SBA	Mean % 2010	1,228	36.1	1,974	44.7	1,760	32.6	0.042	0.395
	Mean % 2018	1,404	64.1	3,399	52.9	4,534	60.7	0.017	0.019
	Mean difference		27.9		8.2		28.1	0.001	0.947
BCG	Mean % 2010	1,429	60.9	816	71.8	247	34.8	0.026	<.0001
	Mean % 2018	1,276	91.6	3,182	76.8	4,480	73.0	<.0001	<.0001
	Mean difference		30.7		5.1		38.2	<.0001	0.340
DPT3/Penta	Mean % 2010	1,400	31.8	793	37.8	240	7.0	0.168	<.0001
	Mean % 2018	1,276	75.4	3,182	60.0	4,480	56.4	<.0001	<.0001
	Mean difference		43.6		22.2		49.4	<.0001	0.013
Measles	Mean % 2010	1,413	39.9	817	47.7	244	20.4	0.076	0.003
	Mean % 2018	1,315	74.6	4,419	64.4	5,332	70.9	0.009	0.315
	Mean difference		34.8		16.8		50.5	0.003	0.039
Care seeking for ARI	Mean % 2010	1,696	57.0	1,041	65.7	212	61.8	0.020	0.546
	Mean % 2018	568	60.2	623	65.2	878	72.5	0.336	0.018
	Mean difference		3.3		0.4		10.6	0.111	0.001
ORT	Mean % 2010	2,059	46.3	1,044	58.6	337	74.0	0.034	<.0001

© 2019 Akseer N et al. *JAMA Network Open*

Indicator	Survey	N	Minimal Conflict	N	Moderate Conflict	N	Severe Conflict	Minimal vs. Moderate p-value	Minimal vs. Severe p-value
	Mean % 2018	730	36.4	1,570	37.7	1,873	41.7	0.743	0.157
	Mean difference		9.9		20.9		32.3	0.578	0.410

© 2019 Akseer N et al. *JAMA Network Open*

eTable 8. Adjusted Percentage Point Change in Key RMNCH Interventions by Conflict Status During 2003-2018 and 2010-2018*

	Minimal						Moderate						Severe						2003-2018		2010-2018	
	MICS 2003 %	MICS 2010 %	2003-2010 AAPC	MICS 2010 %	AHS 2018 %	2010-2018 AAPC†	MICS 2003 %	MICS 2010 %	2003-2010 AAPC	MICS 2010 %	AHS 2018 %	2010-2018 AAPC	MICS 2003 %	MICS 2010 %	2003-2010 AAPC	MICS 2010 %	AHS 2018 %	2010-2018 AAPC‡	Minimal vs. Moderate p-value	Minimal vs. Severe p-value	Minimal vs. Moderate p-value	Minimal vs. Severe p-value
Contraceptive Use	11.0	12.8	0.2	11.9	23.9	1.3	7.1	19.7	1.6	20.7	25.3	0.5	8.4	26.1	2.2	25.0	38.0	1.4	.001	<.001	<.001	.02
ANC	18.0	46.8	3.6	46.5	36.4	-1.1	14.5	49.3	4.4	51.6	28.5	-2.6	8.5	38.9	3.8	40.8	31.0	-1.1	.021	.07	<.001	.97
SBA	16.1	35.9	2.5	36.1	60.4	2.7	12.5	39.3	3.3	41.4	54.6	1.5	8.3	27.4	2.4	29.0	54.1	2.8	.002	.29	.005	.76
BCG	33.9	60.0	3.3	65.7	89.2	2.6	42.7	69.2	3.3	66.7	71.7	0.6	38.3	34.1	-0.5	38.0	74.2	4.0	.85	.002	<.001	.88
DPT3/Penta	29.4	37.5	1.0	37.7	76.0	4.3	23.9	29.8	0.7	33.5	52.8	2.1	21.8	5.4	-2.0	6.2	60.8	6.1	.77	<.0001	<.001	<.0001
Measles	25.5	40.3	1.9	43.0	74.4	3.5	21.1	45.5	3.1	43.6	69.1	2.8	28.9	20.7	-1.0	23.8	62.2	4.3	.04	.01	.44	<.001
ORT	27.2	49.3	2.8	47.5	35.7	-1.3	40.3	53.9	1.7	54.1	42.8	-1.3	7.5	74.9	8.4	73.4	39.3	-3.8	.11	<.001	.89	.004
Care seeking for ARI	47.5	56.8	1.2	54.4	62.2	0.9	40.5	64.0	2.9	65.3	71.6	0.7	54.1	56.1	0.3	59.1	69.2	1.1	.008	.56	.91	.81

Abbreviations: AAPC: Average Annual % Point Change, AHS: Afghanistan Health Survey, ANC: Antenatal care, ARI: Acute respiratory infection, BCG: bacille Calmette-Guerin vaccine, DPT3: diphtheria, pertussis and tetanus (3 doses), MICS: Multiple Indicator Cluster Survey, ORT: Oral rehydration therapy, RMNCH: Reproductive, maternal, newborn and child health, SBA: Skilled birth attendant

*Models adjusted for the main effects of conflict and time, and covariates including maternal illiteracy, contracting type, and rural residence; complete results in the appendix.

† (% in 2010 – % in 2003) / 8 years.

‡ (% in 2018 – % in 2010) / 9 years.

© 2019 Akseer N et al. *JAMA Network Open*

eTable 9. Health Systems Composite Indicators by Conflict Status

A, 2004-2010

Indicator	Survey	N	Minimal Conflict Group	N	Moderate Conflict Group	N	Severe Conflict Group	Minimal vs. Moderate p-value	Minimal vs. Severe p-value
Domain A Client and Community	Mean % 2004	21	60.71(18.01)	8	65.98(6.86)	5	64.68(11.07)	0.690	0.862
	Mean % 2010	21	79.37(7.22)	8	79.48(6.59)	5	67.54(14.38)	0.999	0.021
	Mean difference (SD)		18.66(15.36)		13.5(10.66)		2.86(8.34)	0.693	0.067
Domain B Human Resources	Mean % 2004	20	51.17(7.35)	8	52.86(8.3)	5	56.12(6.81)	0.853	0.397
	Mean % 2010	20	68.77(8.57)	8	73.73(7.08)	5	63.64(16.54)	0.354	0.698
	Mean difference (SD)		17.6(9.99)		20.86(11.46)		7.52(15.29)	0.766	0.186
Domain C Physical Capacity	Mean % 2004	20	46.99(7.87)	8	48.1(6.28)	5	54.82(11.94)	0.944	0.154
	Mean % 2010	20	75.571(8.46)	8	74.18(4.98)	5	67.76(7.98)	0.902	0.123
	Mean difference (SD)		29.33(9.89)		26.08(8.67)		12.94(6.18)	0.678	0.003
Domain D Quality of Service Provision	Mean % 2004	20	48.6(8.24)	8	38.76(8.4)	5	43.56(15.4)	0.050	0.549
	Mean % 2010	20	48.92(10.90)	8	49.69(8.84)	5	38(12.9)	0.966	0.136
	Mean difference (SD)		0.32(13.41)		10.93(7.17)		-5.56(12.15)	0.107	0.598
Domain E Management Systems	Mean % 2004	20	64.97(13.1)	8	70.56(13.47)	5	49.6(29.21)	0.692	0.160
	Mean % 2010	20	78.98(12.97)	8	79.1(12.93)	5	65.16(22.71)	0.999	0.154
	Mean difference (SD)		14.86(20.11)		8.54(24.19)		15.56(17.19)	0.749	0.998
Domain F Overall Mission	Mean % 2004	20	50.48(1.81)	8	49.63(2.15)	5	49.98(0.39)	0.494	0.842
	Mean % 2010	20	50.31(2.68)	8	50.69(3.52)	5	50.58(2.49)	0.948	0.981
	Mean difference (SD)		-0.18(3.78)		1.06(3.69)		0.6(2.75)	0.698	0.905
Overall Means (Provincial)	Mean % 2004	20	52.15(4.19)	8	51.38(3.42)	5	53.62(8.65)	0.925	0.819
	Mean % 2010	20	66.41(6.50)	8	67.89(4.15)	5	58.88(10.05)	0.856	0.074

© 2019 Akseer N et al. *JAMA Network Open*

	Mean difference (SD)		14.9(6.54)		16.51(6.29)		5.26(4.57)	0.812	0.012

B, 2011-2016

Indicator	Survey	N	Minimal Conflict Group	N	Moderate Conflict Group	N	Severe Conflict Group	Minimal vs. Moderate p-value	Minimal vs. Severe p-value
Domain A Client and Community	Mean % 2011	7	78.96(4.55)	15	75.68(8.48)	11	76.29(13.71)	0.757	0.847
	Mean % 2016	7	79.16(6.35)	15	81.25(5.3)	11	79.58(9.45)	0.792	0.992
	Mean difference (SD)		0.2(8.05)		5.23(7.83)		3.29(6.98)	0.331	0.681
Domain B Human Resources	Mean % 2011	7	50.47(6.8)	15	46.51(5.38)	11	52.54(6.28)	0.332	0.758
	Mean % 2016	7	57.97(4.64)	15	54.15(8.85)	11	56.85(6.22)	0.498	0.948
	Mean difference (SD)		7.5(5.23)		7.06(9.41)		4.32(6.81)	0.992	0.685
Domain C Physical Capacity	Mean % 2011	7	33.59(2.75)	15	36.2(8.23)	11	41.35(9.12)	0.747	0.116
	Mean % 2016	7	76.56(4.93)	15	77.95(9.15)	11	77.61(8.58)	0.927	0.963
	Mean difference (SD)		42.97(3.75)		41.37(11.83)		36.26(14.42)	0.952	0.472
Domain D Quality of Service Provision	Mean % 2011	7	46.33(7.69)	15	50.68(8.55)	11	51.2(8.12)	0.489	0.449
	Mean % 2016	7	58.99(9.21)	15	60.54(12.65)	11	60.17(9.09)	0.947	0.973
	Mean difference (SD)		12.66(11.89)		9.46(14.17)		8.97(8)	0.830	0.801
Domain E Management Systems	Mean % 2011	7	42.33(3.9)	15	43.23(8.24)	11	53.95(18.23)	0.985	0.132
	Mean % 2016	7	51.13(14.53)	15	50.35(14.7)	11	54.68(13.35)	0.992	0.865
	Mean difference (SD)		8.8(12.95)		5.53(8.12)		0.74(8.74)	0.733	0.200
Domain F Overall Mission	Mean % 2011	7	46.41(2.47)	15	48.15(3.53)	11	47.48(4.28)	0.553	0.816
	Mean % 2016	7	41.47(7.41)	15	42.6(11.3)	11	50.39(11.06)	0.969	0.200
	Mean difference (SD)		-4.94(6.23)		-5.95(11.15)		2.91(11.27)	0.975	0.277

© 2019 Akseer N et al. *JAMA Network Open*

Overall Means (Provincial)	Mean % 2011	7	54.6(4.29)	15	54.55(4.9)	11	56.53(10.41)	0.999	0.843
	Mean % 2016	7	62.21(4.6)	15	62.38(7.19)	11	65.36(7.65)	0.998	0.620
	Mean difference (SD)		7.61(4.71)		7.24(6.43)		8.84(13.7)	0.996	0.960

© 2019 Akseer N et al. *JAMA Network Open*

eTable 10. Crude and Multivariable Analysis of Conflict Association With Health Systems Indicators
A, From 2004-2010

Equipment functionality index	Bivariate			Main Effect model w/o covariates			Adjusted		
	Coef.	***95% CI***	***p,value***	***Coef.***	***95% CI***	***p,value***	***Coef.***	***95% CI***	***p,value***
**Conflict Type**									
0=Minimal	ref.			ref.			ref.		
1=Moderate	4.91	(2.90,6.91)	<.0001	14.76	(5.10,24.42)	0.003	11.12	(1.57,20.67)	0.022
2=Severe	8.93	(3.40,14.46)	<.0001	70.90	(19.85,122.0)	0.01	62.40	(12.74,112.07)	0.01
**Year**	5.17	(4.76,5.57)	<.0001	5.79	(5.34,6.25)	<.0001	6.03	(5.56,6.49)	0.00
**Conflict Type 1*time**				,3.71	(5.56,-1.86)	<.0001	-3.26	(-5.07, 1.45)	<.0001
**Conflict Type 2*time**				,13.45	(22.71,-4.18)	<.0001	-12.37	(-21.38, 3.36)	0.01
**Patient volume**	37.79	(31.88,43.71)	<.0001				5.75	(-0.82,12.32)	0.086
**Facility Type**									
BHC	ref.						ref.		
CHC	8.40	(6.77,10.03)	<.0001				8.48	(6.90,10.06)	<.0001
Subcenter	12.86	(9.78,15.93)	<.0001				11.50	(8.20,14.81)	<.0001
**Region**									
Central	ref.						ref.		
Central Highlands	3.71	(-0.91,8.32)	0.116				5.43	(1.16,9.70)	0.013
East	1.52	(-1.19,4.22)	0.273				1.44	(-1.15,4.03)	0.276

© 2019 Akseer N et al. *JAMA Network Open*

North	-0.25	(-2.95,2.44)	0.854				-0.14	(-2.68-2.39)	0.912
North East	-3.81	(-6.60,-1.02)	0.007				-3.28	(-5.94,-0.61)	0.02
South	-6.83	(-10.32,-3.34)	<.0001				-8.80	(-12.02,-5.57)	<.0001
South East	-2.52	(-5.33,0.30)	0.08				-3.13	(-5.75,-0.50)	0.02
West	0.34	(-2.50,3.18)	0.816				0.36	(-2.19,2.91)	0.783
**Province Type**									
Contracting In	ref.						ref.		
Contracting Out	-0.64	(-3.45,2.16)	0.653				0.58	(-2.16,3.33)	0.68

Drug availability index	**Bivariate**			**Main Effect model w/o covariates**			**Adjusted**		
	Coef.	***95% CI***	***p-value***	***Coef.***	***95% CI***	***p-value***	***Coef.***	***95% CI***	***p-value***
**Conflict Type**									
0=Minimal	ref.			ref.			ref.		
1=Moderate	-0.35	(3.11,2.41)	0.802	0.59	(-14.6,15.8)	0.939	-2.93	(-18.44,12.59)	0.712
2=Severe	3.10	(4.56,10.75)	0.43	-68.23	(-150,13.4)	0.10	-75.81	(-157.21,5.59)	0.068

© 2019 Akseer N et al. *JAMA Network Open*

**Year**	0.52	(0.14,1.17)	0.122	0.59	(-0.14,1.32)	0.11	0.82	(0.06,1.58)	0.04
**Conflict Type 1*time**				-0.36	(-3.28,2.57)	0.810	0.18	(-2.77,3.14)	0.903
**Conflict Type 2*time**				12.79	(-2.02,27.6)	0.091	13.81	(-0.95,28.57)	0.067
**Patient volume**	-3.80	(11.95,4.35)	0.361				-9.35	(-19.67,0.96)	0.076
**Facility Type**									
BHC	ref.						ref.		
CHC	0.07	(-2.10,2.25)	0.948				0.82	(-1.46,3.10)	0.48
Subcenter	1.89	(-2.25,6.02)	0.371				3.92	(-1.05,8.89)	0.12
**Region**									
Central	ref.						ref.		
Central Highlands	-3.48	(-9.09,2.13)	0.224				-1.96	(-7.92,4.00)	0.52
East	0.91	(-2.45,4.28)	0.595				1.26	(-2.43,4.94)	0.504
North	-2.45	(-5.75,0.84)	0.145				-2.10	(-5.64,1.44)	0.245
North East	-0.19	(-3.64,3.25)	0.912				-0.02	(-3.80,3.77)	0.99

© 2019 Akseer N et al. *JAMA Network Open*

South	-0.74	(-5.05,3.56)	0.735				-0.53	(-5.07,4.00)	0.82
South East	-8.84	(-12.32 5.35)	<.0001				-8.57	(-12.29, 4.86)	<.0001
West	1.91	(-1.58,5.39)	0.284				2.15	(-1.42,5.72)	0.237
**Province Type**									
Contracting In	ref.						ref.		
Contracting Out	-3.10	(-6.55,0.35)	0.078				-1.15	(-4.98,2.69)	0.56

Infrastructure index	**Bivariate**			**Main Effect model w/o covariates**			**Adjusted**		
	Coef.	***95% CI***	***p-value***	***Coef.***	***95% CI***	***p-value***	***Coef.***	***95% CI***	***p-value***
**Conflict Type**									
0=Minimal	ref.			ref.			ref.		
1=Moderate	6.64	(3.96,9.32)	<.0001	12.86	(-0.56,26.28)	0.060	11.78	(-1.42,24.99)	0.08
2=Severe	11.85	(4.47,19.23)	0.002	-38.64	(-108.50,31.22)	0.278	-35.17	(-103.39,33.04)	0.312
**Year**				4.27	(3.65,4.89)	<.0001	3.50	(2.86,4.13)	<.0001

© 2019 Akseer N et al. *JAMA Network Open*

**Conflict Type 1*time**				-2.75	(-5.29,-0.20)	0.035	-2.26	(-4.75,0.24)	0.077
**Conflict Type 2*time**				7.27	(-5.40,19.94)	0.261	6.99	(-5.38,19.36)	0.268
**Patient volume**	68.98	(60.57,77.38)	<.0001				27.52	(18.16,36.88)	<.0001
**Facility Type**									
BHC	ref.						ref.		
CHC	20.20	(17.71,22.69)	<.0001				18.69	(16.21,21.16)	<.0001
Subcenter	36.41	(31.82,40.99)	<.0001				29.54	(24.56,34.53)	<.0001
**Region**									
Central	ref.						ref.		
Central Highlands	-7.68	(-16.22,0.86)	0.078				-7.19	(-14.14,-0.23)	0.043
East	-2.56	(-7.4,2.29)	0.301				-1.06	(-5.22,3.09)	0.616
North	-1.46	(-6.41,3.48)	0.562				2.79	(-1.35,6.92)	0.187
North East	0.24	(-4.79,5.26)	0.926				1.81	(-2.48,6.10)	0.408
South	-1.61	(-7.94,4.72)	0.618				-0.02	(-5.24,5.20)	0.995
South East	-5.22	(-10.27,-0.16)	0.043				-2.97	(-7.21,1.28)	0.171
West	-9.04	(-14.21,-3.86)	0.001				-7.04	(-11.18,-2.89)	0.001
**Province Type**									

© 2019 Akseer N et al. *JAMA Network Open*

Contracting In	ref.						ref.		
Contracting Out	-3.42	(-8.53-1.68)	0.189				-5.95	(-10.43,-1.47)	0.009

Patient history and physical examination	Bivariate			Main Effect model w/o covariates			Adjusted		
	Coef.	***95% CI***	***p-value***	***Coef.***	***95% CI***	***p-value***	***Coef.***	***95% CI***	***p-value***
**Conflict Type**									
0=Minimal	ref.			ref.			ref.		
1=Moderate	-5.18	(-6.94,-3.42)	<.0001	-16.30	(-25.18,-7.43)	<.0001	-12.15	(-21.34,-2.96)	0.01
2=Severe	-4.11	(-9.00,0.77)	0.099	39.33	(-12.57,91.24)	0.137	12.06	(-36.39,60.51)	0.626
**Year**				2.78	(2.32,3.23)	<.0001	1.64	(1.18,2.09)	<.0001
**Conflict Type 1*time**				1.37	(-0.35,3.09)	0.119	0.98	(-0.77,2.74)	0.271
**Conflict Type 2*time**				-8.83	(-18.21,0.56)	0.065	-4.00	(-12.79,4.79)	0.372
**Patient volume**	4.88	(-0.11,9.87)	0.055				0.13	(-5.97,6.23)	0.967
**Facility Type**									

BHC	ref.						ref.		
CHC	0.66	(-0.69,2.00)	0.340				0.82	(-0.45,2.08)	0.206
Subcenter	-1.29	(-3.83,1.25)	0.321				0.45	(-2.38,3.29)	0.754
**Region**									
Central	ref.						ref.		
Central Highlands	-6.14	(-9.35,-2.93)	<.0001				-3.43	(-6.66,-0.21)	0.037
East	2.11	(0.13,4.08)	0.037				2.57	(0.55,4.60)	0.013
North	-0.89	(-2.79,1.01)	0.357				-1.54	(-3.47,0.38)	0.115
North East	-7.36	(-9.36,-5.35)	<.0001				-5.94	(-8.01,-3.86)	0
South	-5.94	(-8.46,-3.42)	<.0001				-8.02	(-10.49,-5.55)	0
South East	-3.06	(-5.11,-1.01)	0.003				-4.42	(-6.46,-2.39)	0
West	2.10	(0.07-4.13)	0.042				0.50	(-1.44,-2.44)	0.614
**Province Type**									
Contracting In	ref.						ref.		
Contracting Out	-4.85	(-6.9,-2.79)	<.0001				-2.68	(-4.76,-0.60)	0.012

© 2019 Akseer N et al. *JAMA Network Open*

Patient counseling index	Bivariate			Main Effect model w/o covariates			Adjusted		
	Coef.	*95% CI*	*p-value*	*Coef.*	*95% CI*	*p-value*	*Coef.*	*95% CI*	*p-value*
**Conflict Type**									
0=Minimal	ref.			ref.			ref.		
1=Moderate	-6.44	(-8.75,-4.13)	<.0001	-9.83	(-21.68,2.03)	0.104	-0.49	(-13.69,12.71)	0.942
2=Severe	-8.04	(-14.43,-1.65)	0.014	-27.14	(-96.25,41.97)	0.441	-47.32	(-116.89,22.25)	0.182
**Year**				1.49	(0.88,2.09)	<.0001	0.81	(0.15,1.47)	0.016
**Conflict Type 1*time**				0.22	(-2.07,2.51)	0.850	-0.92	(-3.44,1.60)	0.474
**Conflict Type 2*time**				2.95	(-9.54,15.45)	0.643	6.85	(-5.77,19.46)	0.288
**Patient volume**	3.99	(-3.07-11.04)	0.268				1.59	(-7.18,10.37)	0.722
**Facility Type**									
BHC	ref.						ref.		
CHC	0.42	(-1.39-2.23)	0.648				0.71	(-1.12,2.53)	0.45
Subcenter	-0.95	(-4.35-2.44)	0.582				-0.65	(-4.73,3.43)	0.75
**Region**									
Central	ref.						ref.		

© 2019 Akseer N et al. *JAMA Network Open*

Central Highlands	-17.81	(-22.2,-13.41)	<.0001				-14.99	(-19.65,-10.33)	0
East	-4.26	(-6.93,-1.58)	0.002				-2.47	(-5.39,0.46)	0.098
North	-3.31	(-5.9,-0.72)	0.012				-2.59	(-5.37,0.19)	0.068
North East	-8.93	(-11.66,-6.21)	<.0001				-6.87	(-9.87,3.88)	0
South	-10.56	(-13.99,-7.13)	<.0001				-10.76	(-14.32,-7.19)	0
South East	-7.59	(-10.37,-4.8)	<.0001				-7.31	(-10.25,-4.37)	0
West	-2.82	(-5.58,-0.05)	0.046				-3.29	(-6.10,-0.48)	0.022
**Province Type**	-106.19	(-188.38, -24.01)	0.011						
Contracting In	ref.						ref.		
Contracting Out	-9.34	(-12.07,-6.62)	<.0001				-5.19	(-8.20,-2.18)	0.001

B, From 2011-2016

Equipment Functionality Index	**Bivariate**			**Main Effect model w/o covariates**			**Adjusted**		
	Coef.	***95% CI***	***p-value***	***Coef.***	***95% CI***	***p-value***	***Coef.***	***95% CI***	***p-value***
**Conflict Type**									

© 2019 Akseer N et al. *JAMA Network Open*

0=Minimal	ref.			ref.			ref.		
1=Moderate	6.8	(4.6,9.1)	<.0001	6.4	(3.0,9.7)	<.0001	3.6	(0.5,6.8)	0.025
2=Severe	7.9	(5.8,10.0)	<.0001	10.1	(6.9,13.2)	<.0001	4.2	(1.2,7.2)	0.007
**Year**	2.3	(2.1,2.5)	<.0001	2.5	(2.1,2.9)	<.0001	2.5	(2.0,2.9)	<.0001
**Conflict Type 1*time**	--		--	0.1	(-0.4,0.6)	0.720	0.1	(-0.4,0.6)	0.588
**Conflict Type 2*time**	--		--	-0.4	(-0.9,0.1)	0.111	-0.4	(-0.9,0.1)	0.105
**Patient volume (10,000s)**	-0.1	(-0.2,-0.1)	<.0001				-0.2	(-0.2,-0.1)	<.0001
**Facility Type**									
BHC	ref.						ref.		
CHC	4.4	(2.9,5.9)	<.0001				4	(2.7,5.3)	<.0001
Subcenter	-14.4	(-15.9,-12.9)	<.0001				-13.7	(-15.1,-12.3)	<.0001
**Distance of facility in KM from provincial center (1000s)**	-9.6	(-14.0,-5.3)	0.001				-5.6	(-9.4,-1.8)	0.004
**Region**									
Central	ref.						ref.		
Central Highlands	6.3	(3.1,9.6)	<.0001				2.7	(0.0,5.5)	0.049
East	-1.2	(-3.7,1.4)	0.374				-1.7	(-3.9,0.5)	0.138
North	0.1	(-2.6,2.8)	0.937				-2.8	(-5.0,-0.6)	0.013
North East	-0.2	(-2.8,2.3)	0.851				-0.5	(-2.7,1.7)	0.650
South	-7.6	(-10.1,-5.0)	<.0001				-7.6	(-9.9,-5.4)	<.0001
South East	1.1	(-1.6,3.8)	0.414				-0.3	(-2.7,2.0)	0.775
West	-3.2	(-5.7,-0.8)	0.01				-2.9	(-4.9,-0.9)	0.004
**Province Type**									
Contracting In	ref.						ref.		
Contracting Out	8.4	(5.9,11.0)	<.0001				7.7	(5.1,10.2)	<.0001

Pharmaceuticals and Vaccines Availability Index	**Bivariate**	**Main Effect model w/o covariates**	**Adjusted**

© 2019 Akseer N et al. *JAMA Network Open*

	Coef.	*95% CI*	*p-value*	*Coef.*	*95% CI*	*p-value*	*Coef.*	*95% CI*	*p-value*
**Conflict Type**									
0=Minimal	ref.			ref.			ref.		
1=Moderate	10.7	(8.4,13.0)	<.0001	1.6	(-2.1,5.4)	0.393	-5.0	(-8.5,-1.5)	0.005
2=Severe	12.5	(10.3,14.7)	<.0001	5.5	(1.9,9.0)	<.0001	-4.7	(-8.1,-1.4)	0.005
**Year**	0.3	(0.1,0.5)	0.010	-1.1	(-1.6,-0.6)	<.0001	-1.1	(-1.6,-0.6)	<.0001
**Conflict Type 1*time**	--		--	1.9	(1.3,2.4)	<.0001	1.9	(1.3,2.4)	<.0001
**Conflict Type 2*time**	--		--	1.4	(0.9,2.0)	<.0001	1.5	(0.9,2.0)	<.0001
**Patient volume (10,000s)**	-0.3	(-0.3,-0.2)	<.0001				-0.3	(-0.3,-0.2)	<.0001
**Facility Type**									
BHC	ref						ref.		
CHC	6.6	(5.0,8.2)	<.0001				6.4	(5.0,7.7)	<.0001
Subcenter	-12.2	(-13.8,-10.5)	<.0001				-11.4	(-12.8,-10.0)	<.0001
**Distance of facility in KM from provincial center (1000s)**	-6.4	(-11.0,-1.7)	0.007				-3.6	(-7.9,0.7)	0.100
**Region**									
Central	ref						ref.		
Central Highlands	10.2	(6.8,13.6)	<.0001				2.5	(-0.3,5.2)	0.077
East	2.6	(0.0,5.3)	0.054				-1.9	(-4.1,0.4)	0.103
North	7.1	(4.2,9.9)	<.0001				0.6	(-1.6,2.9)	0.581
North East	4.0	(1.3,6.6)	0.004				-0.4	(-2.7,1.8)	0.730
South	-3.2	(-5.9,-0.5)	0.020				-6.9	(-9.2,-4.7)	<.0001
South East	4.8	(2.0,7.7)	0.001				-0.9	(-3.4,1.5)	0.442
West	1.0	(-1.6,3.5)	0.463				-0.9	(-2.9,1.1)	0.368
**Province Type**									
Contracting In	ref.						ref.		
Contracting Out	19.4	(16.9,21.9)	<.0001				18.1	(16.9,21.9)	<.0001

© 2019 Akseer N et al. *JAMA Network Open*

Functional infrastructure index	Bivariate			Main Effect model w/o covariates			Adjusted		
	Coef.	*95% CI*	*p-value*	*Coef.*	*95% CI*	*p-value*	*Coef.*	*95% CI*	*p-value*
**Conflict Type**									
0=Minimal	ref.								
1=Moderate	4.8	(1.1,8.6)	0.011	6.4	(0.8,12.0)	0.025	8.0	(2.3,13.6)	0.006
2=Severe	7.8	(4.3,11.3)	<0.001	15.3	(10.0,20.6)	<.0001	13.9	(8.5,19.4)	<.0001
**Year**	1.0	(0.8,1.3)	<0.001	1.9	(1.2,2.6)	<.0001	2.0	(1.3,2.7)	<0.001
**Conflict Type 1*time**	--		--	-0.3	(-1.2,0.5)	0.464	-0.4	(-1.2,0.5)	0.408
**Conflict Type 2*time**	--		--	-1.5	(-2.3,-0.7)	<.0001	-1.6	(-2.4,-0.8)	<.0001
**Patient volume (10,000s)**	-0.1	(-0.2,0.0)	0.006				-0.1	(-0.2,0.0)	0.007
**Facility Type**									
BHC	ref.						ref.		
CHC	6.2	(3.5,8.9)	<.0001				5.5	(2.9,8.1)	<.0001
Subcenter	-12.1	(-14.9,-9.4)	<.0001				-10.9	(-13.5,-8.3)	<.0001
**Distance of facility in KM from provincial center (1000s)**	-22.3	(-29.0,-15.6)	<.0001				-14.6	(-21.2,-8.0)	<.0001
**Region**									
Central	ref.						ref.		
Central Highlands	1.7	(-3.5,7.0)	0.517				-0.1	(-5.5,5.3)	0.969
East	-15.7	(-19.8,-11.6)	<.0001				-12.7	(-17.0,-8.4)	<.0001
North	-8.8	(-13.1,-4.5)	<.0001				-9.0	(-13.4,-4.7)	<.0001
North East	-0.6	(-4.7,3.5)	0.765				2.2	(-2.1,6.6)	0.320
South	-19.6	(-23.8,-15.5)	<.0001				-15.5	(-19.9,-11.2)	<.0001
South East	-7.6	(-11.9,-3.3)	0.001				-7.2	(-11.8,-2.6)	0.002
West	-12.5	(-16.4,-8.5)	<.0001				-10.3	(-14.1,-6.4)	<.0001
**Province Type**									
Contracting In	ref.						ref.		
Contracting Out	-3.8	(-8.0,0.5)	0.081				-2.9	(-7.9,2.1)	0.250

© 2019 Akseer N et al. *JAMA Network Open*

Client Background and Physical Assessment Index	Bivariate			Main Effect model w/o covariates			Adjusted		
	Coef.	*95% CI*	*p-value*	*Coef.*	*95% CI*	*p-value*	*Coef.*	*95% CI*	*p-value*
**Conflict Type**									
0=Minimal	ref.			ref.			ref.		
1=Moderate	5.2	(3.8,6.7)	0.04	3.5	(0.6,6.5)	0.019	5	(2.0,8.0)	0.001
2=Severe	3.7	(2.4,5.1)	<.0001	8.8	(6.0,11.6)	<.0001	9.3	(6.4,12.2)	<.0001
**Year**	0.8	(0.6,1.0)	<.0001	1.2	(0.8,1.6)	<.0001	1.2	(0.8,1.7)	<.0001
**Conflict Type 1*time**	--		--	0.3	(-0.2,0.9)	0.200	0.4	(-0.2,0.9)	0.18
**Conflict Type 2*time**	--		--	-1	(-1.5,-0.5)	<.0001	-1.0	(-1.5,-0.5)	<.0001
**Patient volume (10,000s)**	<0.1	(0.0,0.1)	0.004				<0.1	(-0.1,0.0)	0.938
**Facility Type**									
BHC	ref.						ref.		
CHC	1.3	(0.1,2.5)	0.028				1.4	(0.3,2.5)	0.01
Subcenter	0.1	(-1.1,1.3)	0.898				0.4	(-0.7,1.6)	0.48
**Distance of facility in KM from provincial center (1000s)**	1.4	(-2.4,5.2)	0.47				5.9	(2.1,9.7)	0.002
**Region**									
Central	ref						ref.		
Central Highlands	2.2	(0.2,4.3)	0.033				3.9	(1.7,6.1)	0.001
East	-0.2	(-1.9,1.5)	0.797				1.8	(0.0,3.6)	0.057
North	-0.1	(-1.8,1.7)	0.925				0.4	(-1.4,2.2)	0.68
North East	-0.5	(-2.2,1.1)	0.54				1.8	(0.0,3.6)	0.06
South	-5.4	(-7.1,-3.7)	<.0001				-4.8	(-6.6,-3.0)	<.0001
South East	-1.5	(-3.3,0.3)	0.108				0.1	(-1.9,2.0)	0.956
West	-2.1	(-3.7,-0.5)	0.011				-2.0	(-3.6,-0.4)	0.016
**Province Type**									
Contracting In	ref.						ref.		
Contracting Out	-0.3	(-1.9,1.4)	0.75				-3.4	(-5.4,-1.4)	0.001

© 2019 Akseer N et al. *JAMA Network Open*

Client Counseling Index	Bivariate			Main Effect model w/o covariates			Adjusted		
	Coef.	*95% CI*	*p-value*	*Coef.*	*95% CI*	*p-value*	*Coef.*	*95% CI*	*p-value*
**Conflict Type**									
0=Minimal	ref			ref			ref		
1=Moderate	8.2	(5.9,10.5)	<.0001	-2.7	(-7.5,2.1)	0.272	-0.4	(-5.3,4.6)	0.881
2=Severe	6.2	(4.0,8.4)	<.0001	5.7	(1.2,10.2)	0.010	7.0	(2.3,11.7)	<.0001
**Year**	2.8	(2.5,3.1)	<.0001	2	(1.3,2.7)	<.0001	2.0	(1.3,2.7)	<.0001
**Conflict Type 1*time**	--		--	2.2	(1.4,3.1)	<.0001	2.2	(1.4,3.1)	<.0001
**Conflict Type 2*time**	--		--	0.2	(-0.6,1.0)	0.670	0.1	(-0.7,0.9)	0.733
**Patient volume (10,000s)**	0.1	(0.0,0.2)	0.049				0.1	(0.0,0.1)	0.123
**Facility Type**									
BHC	ref						ref.		
CHC	1.0	(-0.9,2.8)	0.305				0.9	(-0.9,2.6)	0.330
Subcenter	-0.6	(-2.6,1.3)	0.520				-0.2	(-2.0,1.6)	0.840
**Distance of facility in KM from provincial center (1000s)**	5.1	(-1.1,11.4)	0.108				11.7	(5.5,17.8)	<.0001
**Region**									
Central	ref						ref.		
Central Highlands	6.9	(3.7,10.2)	<.0001				9.8	(6.4,13.2)	<.0001
East	-1.4	(-4.1,1.3)	0.309				2.4	(-0.5,5.3)	0.107
North	1.0	(-1.8,3.8)	0.472				2.3	(-0.5,5.1)	0.107
North East	-1.9	(-4.6,0.7)	0.151				2.1	(-0.7,5.0)	0.150
South	-4.9	(-7.6,-2.3)	<.0001				-3.8	(-6.7,-0.9)	0.010
South East	-2.5	(-5.4,0.4)	0.086				0.1	(-3.0,3.2)	0.944
West	-2.7	(-5.3,-0.2)	0.037				-1.9	(-4.4,0.6)	0.145
**Province Type**									
Contracting In	ref.						ref.		
Contracting Out	-0.9	(-3.5,1.8)	0.523				-7.0	(-10.2,-3.8)	<.0001

© 2019 Akseer N et al. *JAMA Network Open*

Female health worker Index	Bivariate			Main Effect model w/o covariates			Adjusted		
	Coef.	*95% CI*	*p-value*	*Coef.*	*95% CI*	*p-value*	*Coef.*	*95% CI*	*p-value*
**Conflict Type**									
0=Minimal	ref			ref			ref		
1=Moderate	-2.4	(-5.0,0.1)	0.060	-4.4	(-8.5,-0.3)	0.036	-6.0	(-10.3,-1.8)	0.010
2=Severe	-0.8	(-3.2,1.6)	0.520	-1.8	(-5.6,2.1)	0.370	-4.4	(-8.5,-0.3)	0.034
**Year**	0.9	(0.6,1.1)	<.0001	0.6	(0.1,1.2)	0.030	0.6	(0.1,1.2)	0.030
**Conflict Type 1*time**	--		--	0.4	(-0.3,1.1)	0.235	0.4	(-0.3,1.1)	0.232
**Conflict Type 2*time**	--		--	0.2	(-0.4,0.8)	0.501	0.2	(-0.4,0.8)	0.475
**Patient volume (10,000s)**	<0.01	(0.0,0.1)	0.285				<0.01	(0.0,0.1)	0.159
**Facility Type**									
BHC	ref						ref.		
CHC	5.0	(3.1,6.9)	<.0001				4.9	(3.1,6.8)	<.0001
Subcenter	9.2	(7.3,11.1)	<.0001				9.5	(7.6,11.4)	<.0001
**Distance of facility in KM from provincial center (1000s)**	-2.3	(-7.3,2.8)	0.378				-3.5	(-8.5,1.6)	0.182
**Region**									
Central	ref						ref.		
Central Highlands	-0.5	(-4.2,3.2)	0.797				-1.4	(-5.1,2.4)	0.475
East	-0.9	(-3.8,2.0)	0.557				-2.8	(-5.8,0.3)	0.075
North	3.8	(0.8,6.9)	0.014				3.6	(0.5,6.6)	0.021
North East	5.8	(2.9,8.6)	<.0001				3.5	(0.5,6.6)	0.020
South	0.8	(-2.1,3.7)	0.576				-1.7	(-4.8,1.3)	0.270
South East	5.7	(2.7,8.8)	<.0001				4.8	(1.6,8.1)	0.004
West	2.1	(-0.7,4.8)	0.143				1.0	(-1.8,3.7)	0.487
**Province Type**									
Contracting In	ref.						ref.		

© 2019 Akseer N et al. *JAMA Network Open*

Contracting Out	2.7	(-0.2,5.7)	0.065				4.4	(0.9,7.9)	0.010

Provider knowledge score	**Bivariate**			**Main Effect model w/o covariates**			**Adjusted**		
	Coef.	***95% CI***	***p-value***	***Coef.***	***95% CI***	***p-value***	***Coef.***	***95% CI***	***p-value***
**Conflict Type**									
0=Minimal	ref			ref			ref		
1=Moderate	-2.9	(-4.6,-1.1)	0.001	-7.3	(-10.9,-3.8)	<.0001	-9.2	(-12.7,-5.6)	<.0001
2=Severe	-1.7	(-3.3,0.0)	0.045	-7.5	(-10.8,-4.2)	<.0001	-11.7	(-15.0,-8.3)	<.0001
**Year**	1.3	(1.1,1.6)	<.0001	0.4	(-0.1,0.9)	0.121	0.5	(0.0,1.0)	0.072
**Conflict Type 1*time**	--		--	0.9	(0.3,1.5)	0.005	1.0	(0.3,1.6)	0.003
**Conflict Type 2*time**	--		--	1.2	(0.6,1.8)	<.0001	1.2	(0.6,1.8)	<.0001
**Patient volume (10,000s)**	<0.1	(-0.1,0.0)	0.229				<0.1	(-0.1,0.0)	0.143
**Facility Type**									
BHC	ref						ref.		
CHC	-0.1	(-1.4,1.3)	0.936				<0.1	(-1.3,1.3)	0.99
Subcenter	-5.4	(-6.8,-4.1)	<.0001				-5.1	(-6.4,-3.9)	<.0001
**Distance of facility in KM from provincial center (1000s)**	-3.7	(-7.4,0.1)	0.055				-1.4	(-5.1,2.2)	0.436
**Region**									
Central	ref						ref.		
Central Highlands	1.4	(-1.0,3.8)	0.262				1.7	(-0.8,4.1)	0.187
East	-1.9	(-3.8,0.1)	0.06				-2.6	(-4.7,-0.5)	0.013
North	0.5	(-1.5,2.6)	0.596				0.8	(-1.2,2.8)	0.44
North East	-0.3	(-2.2,1.6)	0.776				-0.7	(-2.7,1.4)	0.53
South	-5.8	(-7.8,-3.9)	<.0001				-6.4	(-8.4,-4.4)	<.0001
South East	4.7	(2.5,6.8)	<.0001				5.4	(3.1,7.6)	<.0001
West	-3.3	(-5.2,-1.4)	0.001				-3.3	(-5.1,-1.5)	<.0001
**Province Type**									

© 2019 Akseer N et al. *JAMA Network Open*

Contracting In	ref.						ref.		
Contracting Out	<0.1	(-2.0,2.0)	0.983				3.2	(0.9,5.5)	0.01

© 2019 Akseer N et al. *JAMA Network Open*

eFigure 1. Average Annual Percentage Point Change in Composite Coverage Index vs Conflict by Province

A, 2003-2010

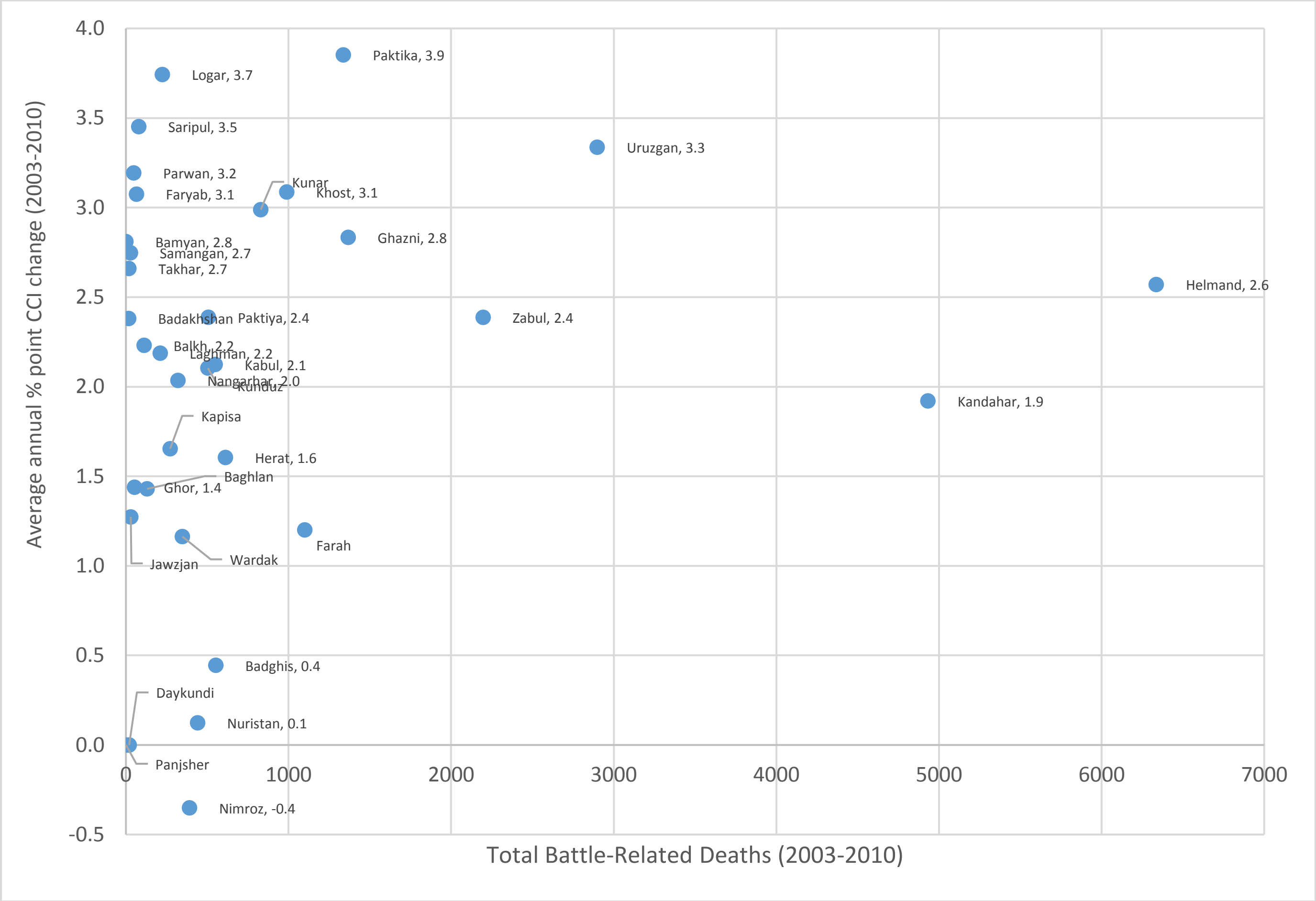

© 2019 Akseer N et al. *JAMA Network Open*

B, 2010-2017

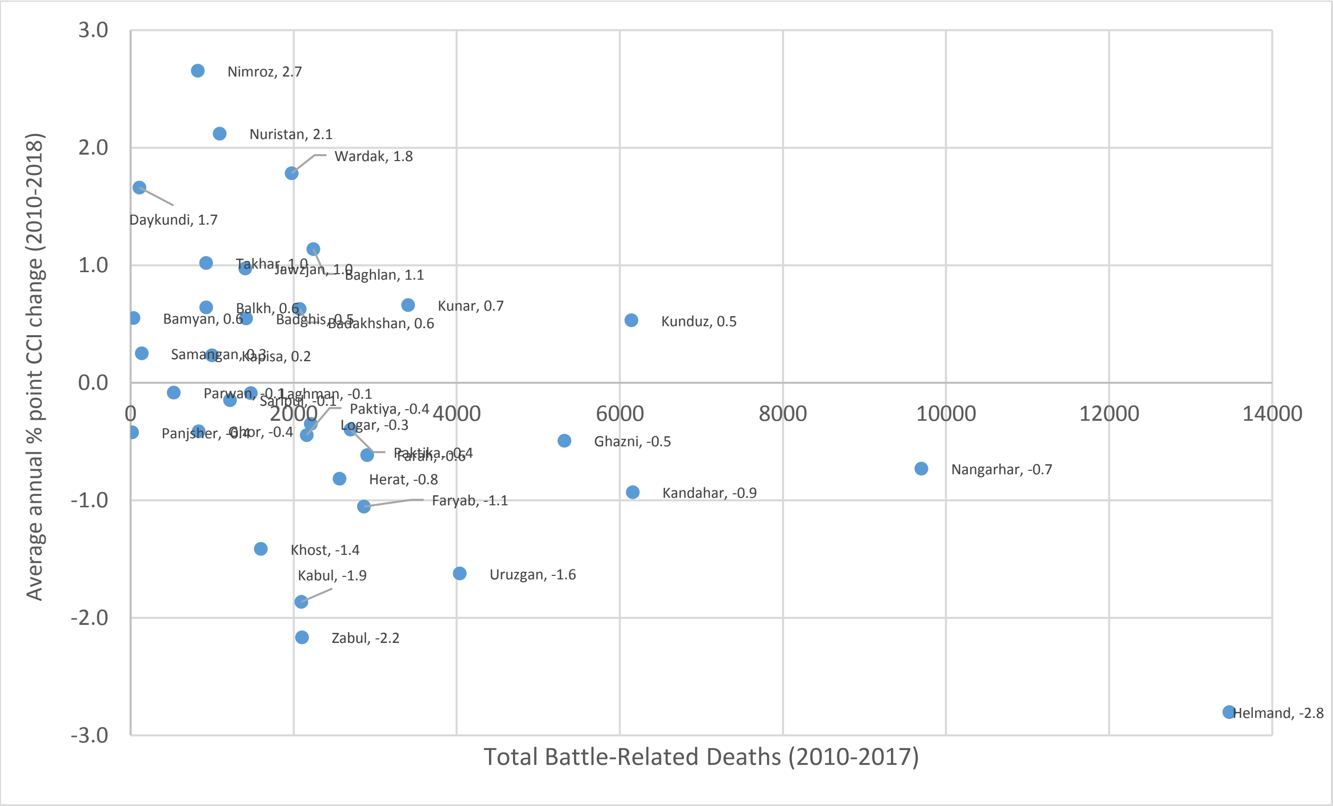

© 2019 Akseer N et al. *JAMA Network Open*

eFigure 2. Crude and Multivariable Analysis of Conflict Association With RMNCH Coverage

A, 2003-2010

* Crude means over time   *Adjusted means over time

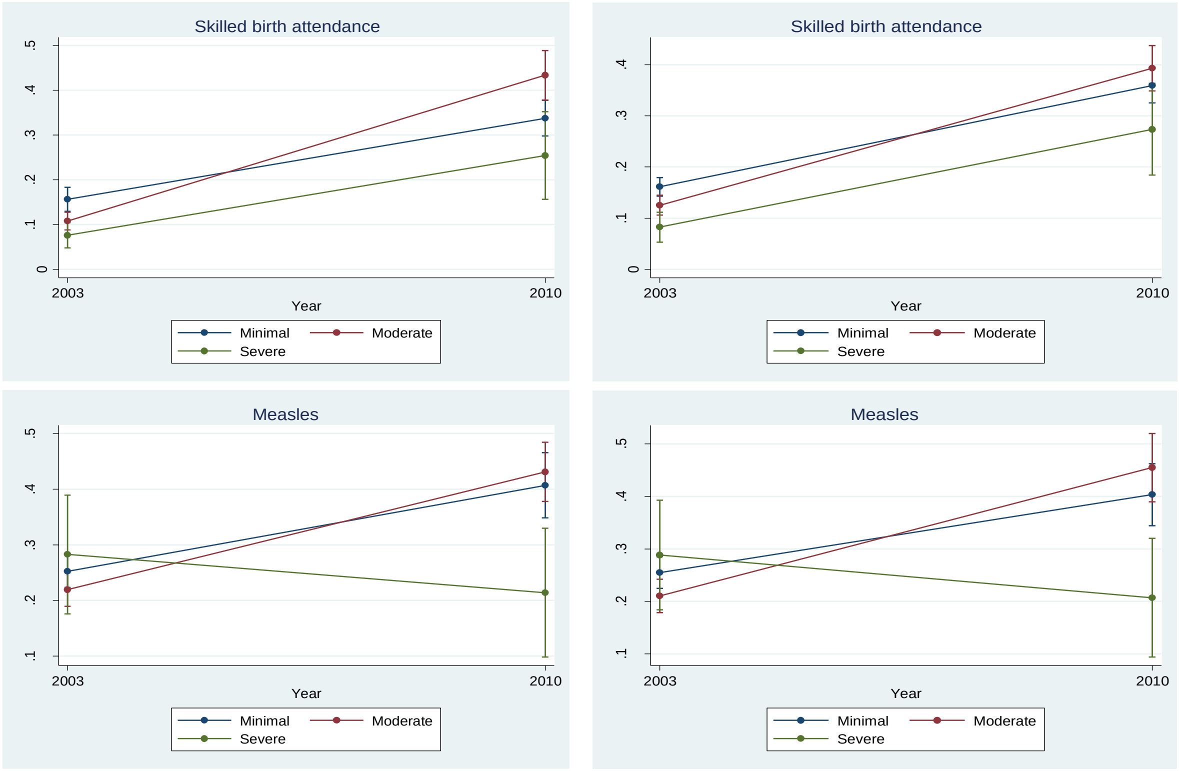

© 2019 Akseer N et al. *JAMA Network Open*

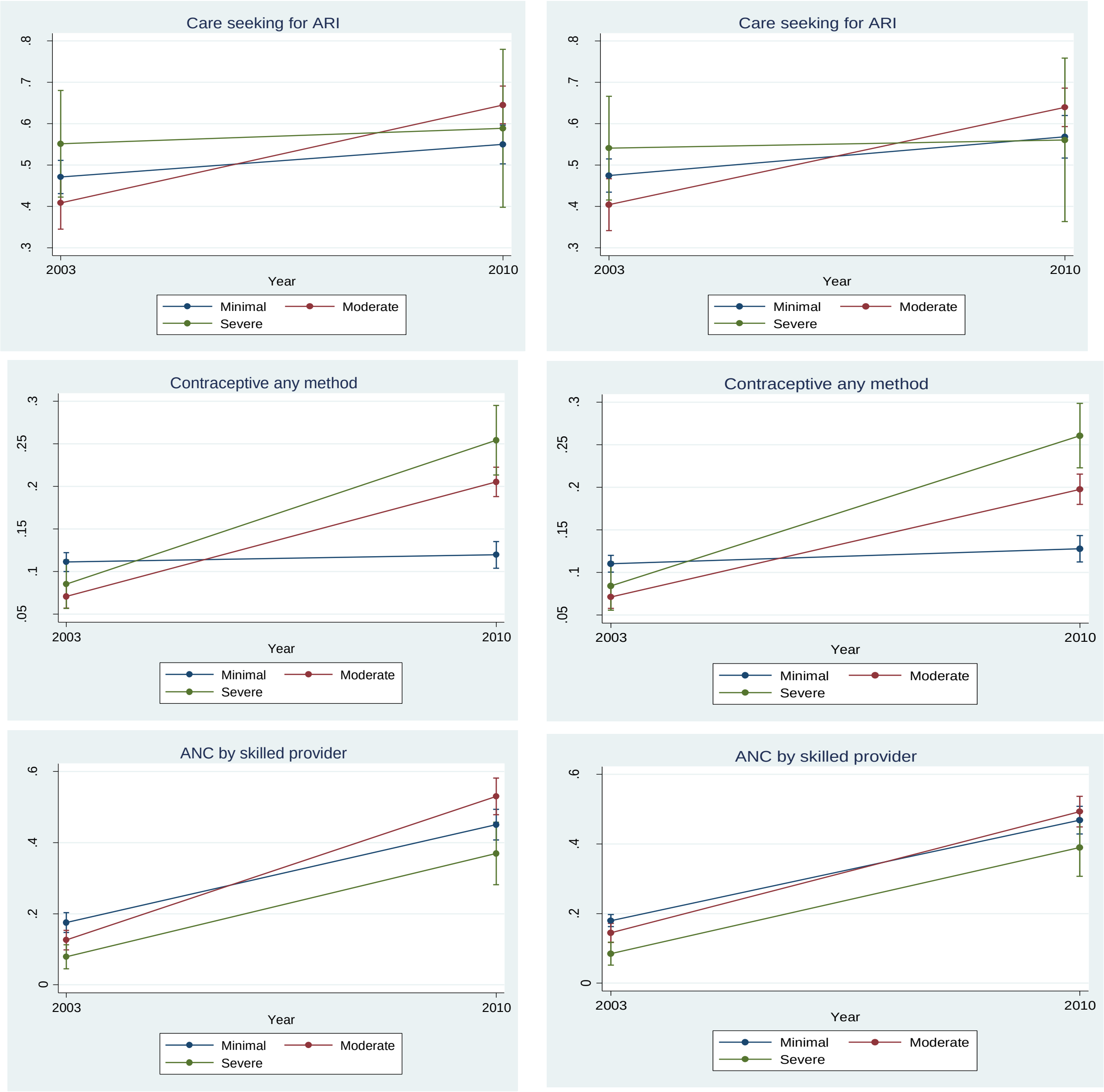

© 2019 Akseer N et al. *JAMA Network Open*

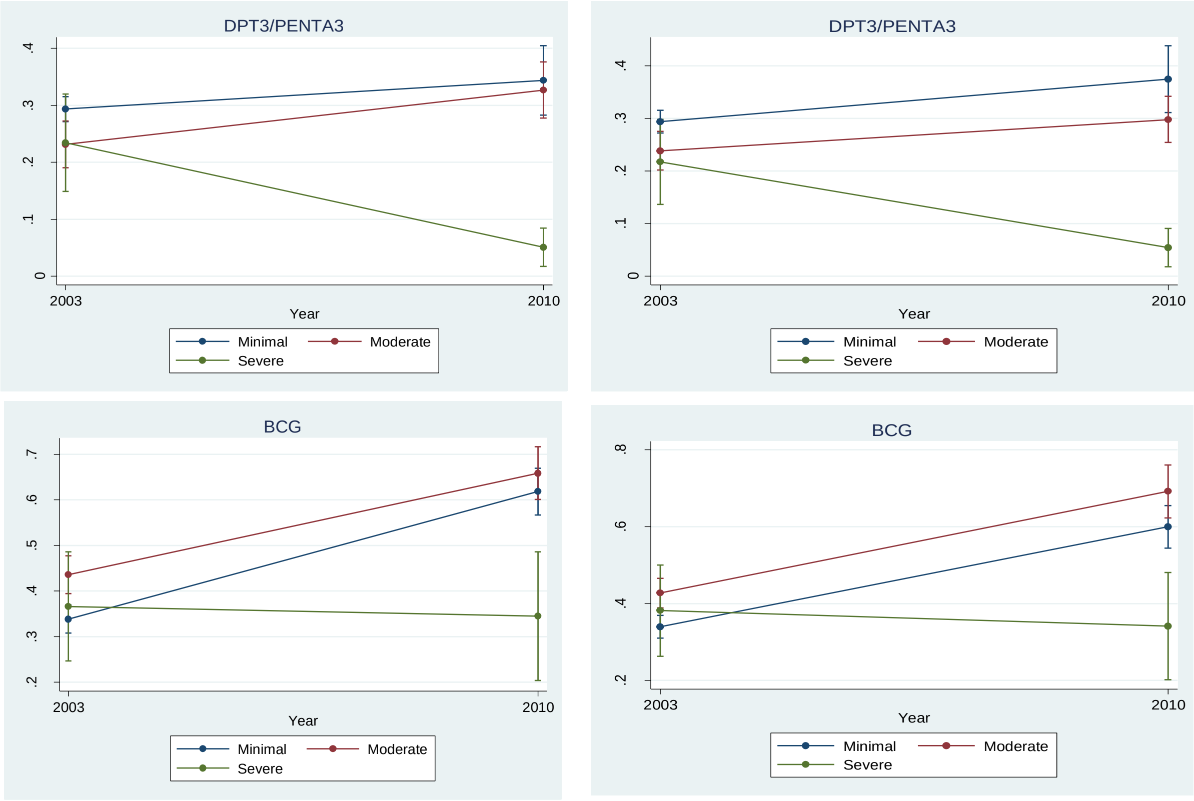

© 2019 Akseer N et al. *JAMA Network Open*

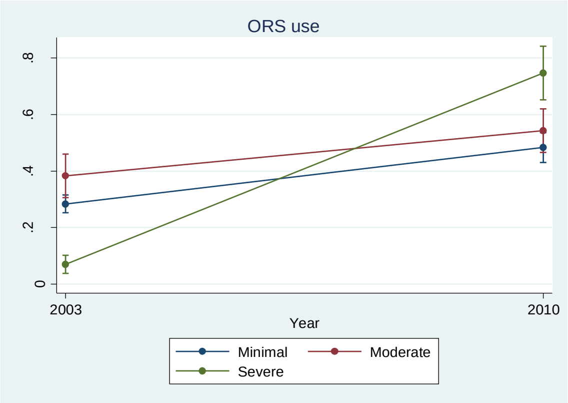

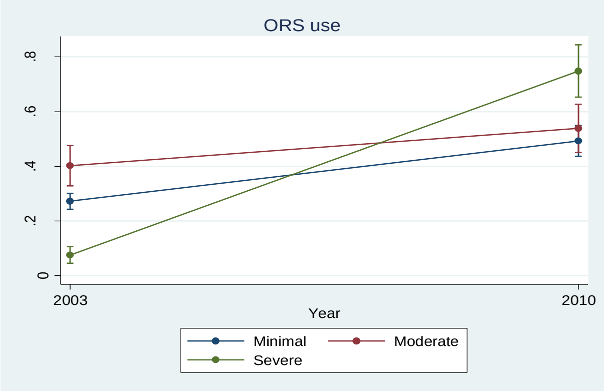

B, 2010-2018

*Unadjusted trends over time

*Covariate adjusted means over time

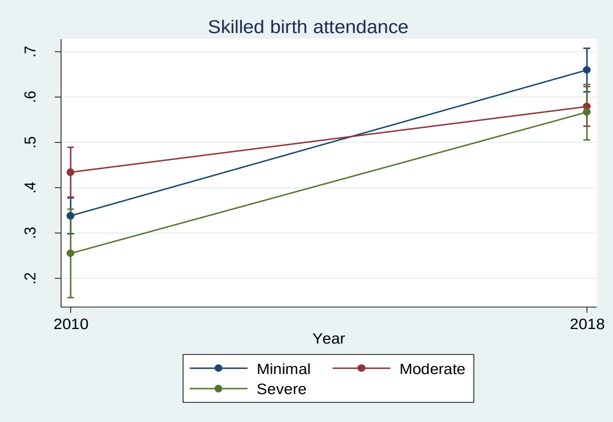

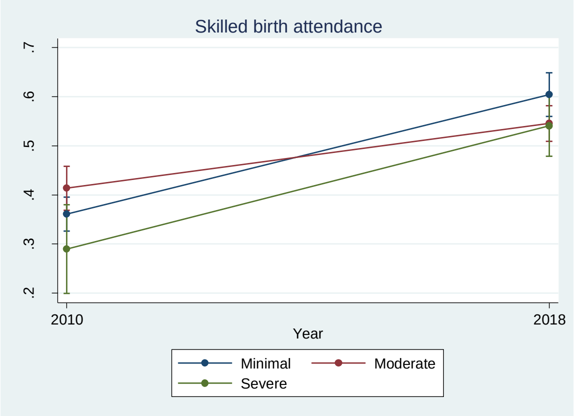

© 2019 Akseer N et al. *JAMA Network Open*

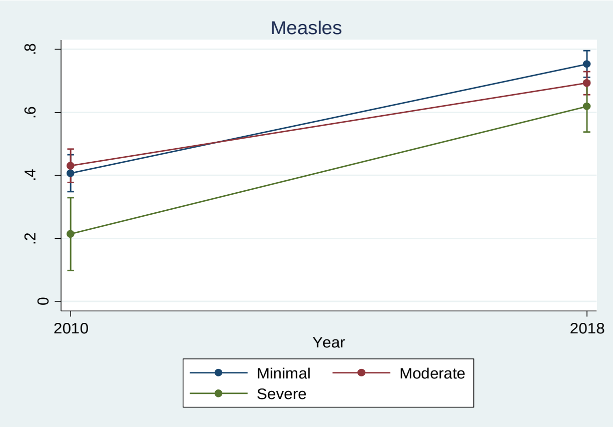

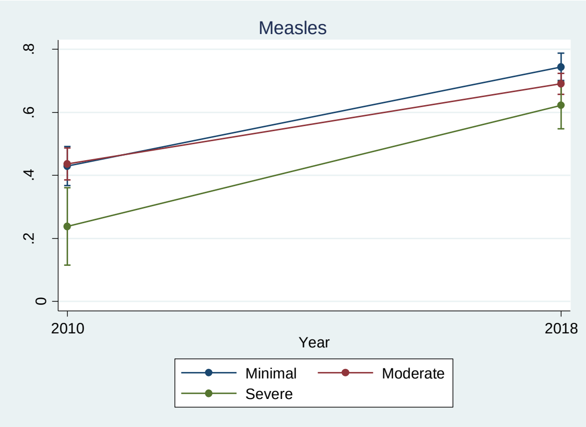

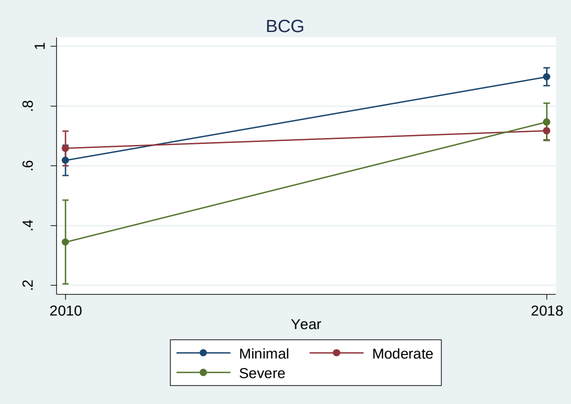

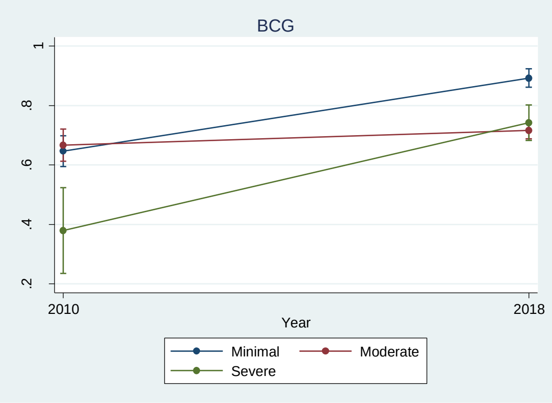

© 2019 Akseer N et al. *JAMA Network Open*

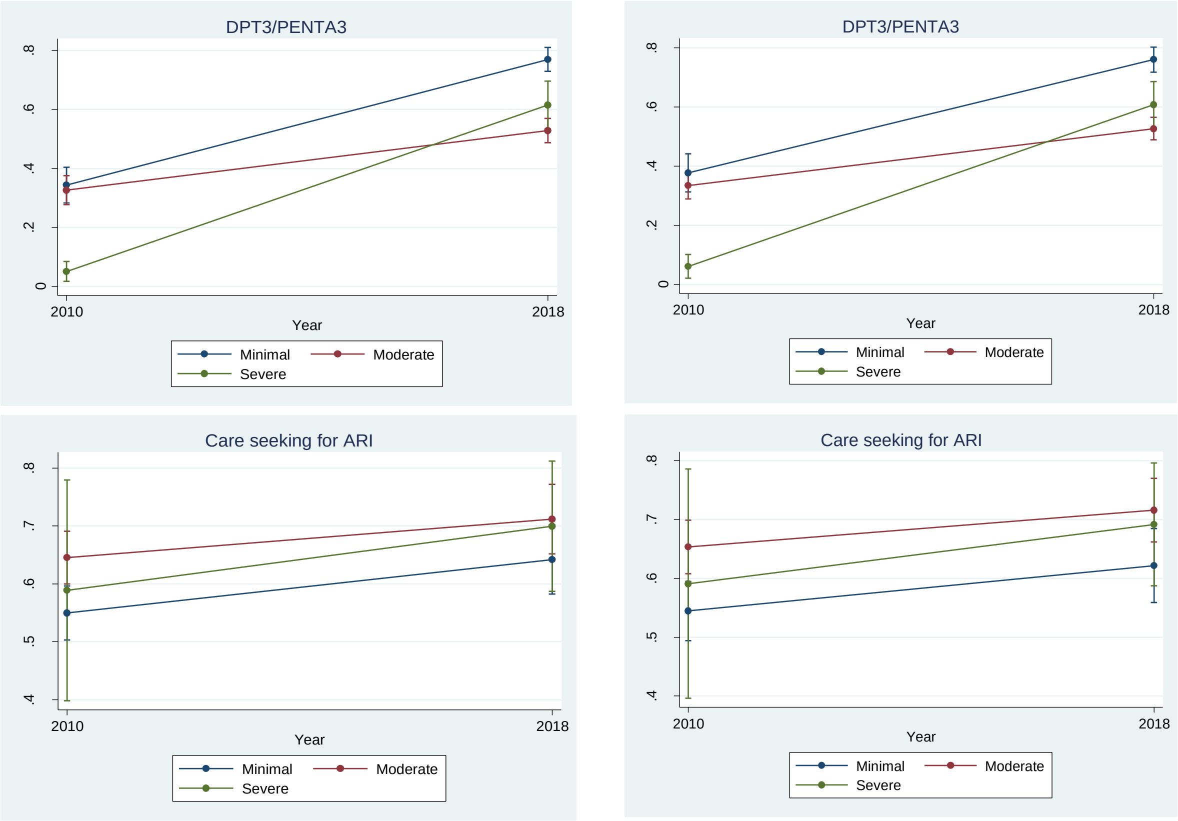

© 2019 Akseer N et al. *JAMA Network Open*

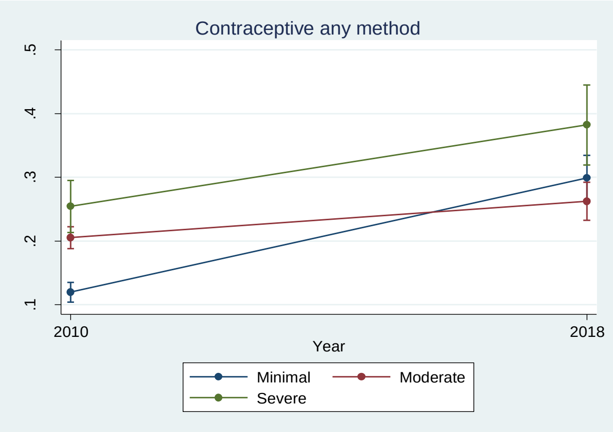

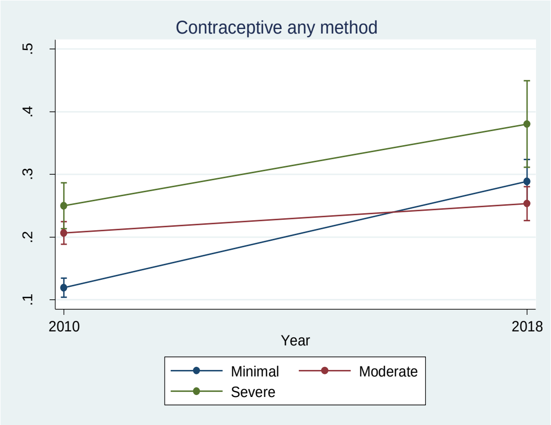

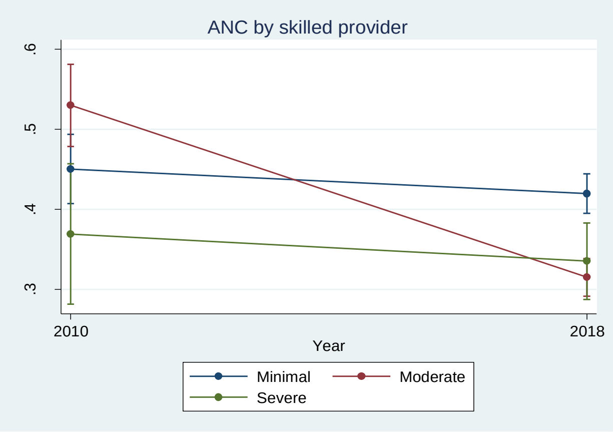

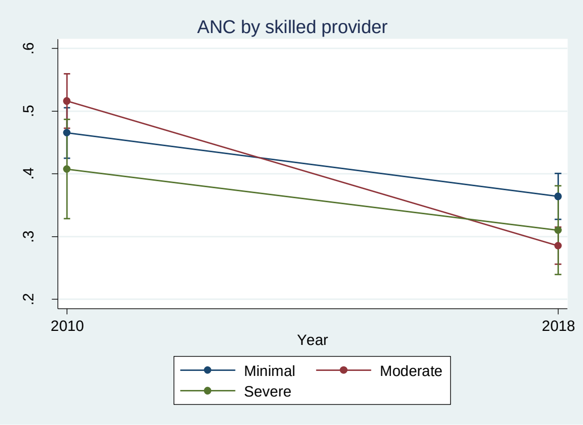

© 2019 Akseer N et al. *JAMA Network Open*

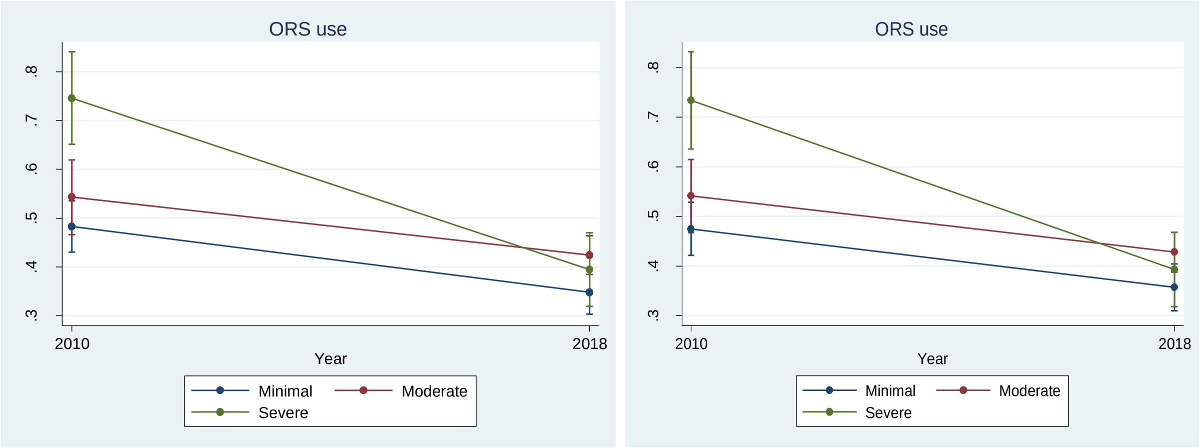

© 2019 Akseer N et al. *JAMA Network Open*

eFigure 3. Health System Overall Mean Scores and Change by Province
A. **2004-2010**

Note: A single point is presented for provinces with an estimate for only that year. The vertical line between them represents percentage point change between years.

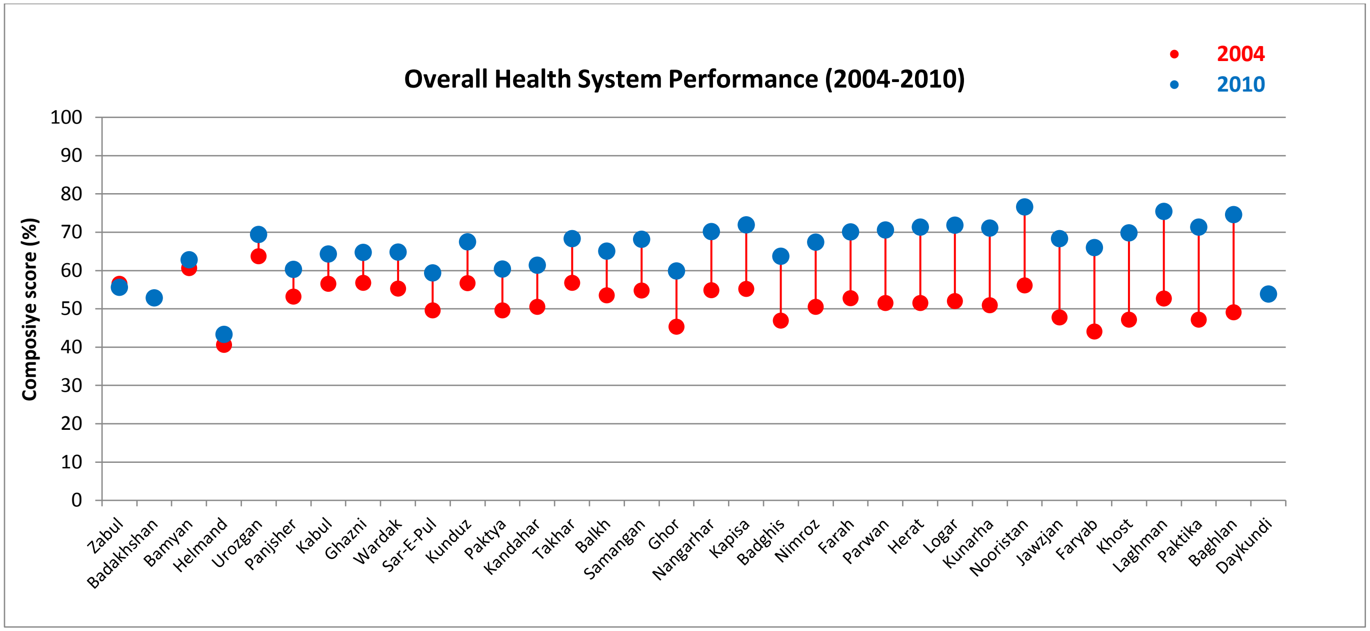

© 2019 Akseer N et al. *JAMA Network Open*

B, 2011-2016

Note: A single point is presented for provinces with an estimate for only that year. The vertical line between them represents percentage point change between years.

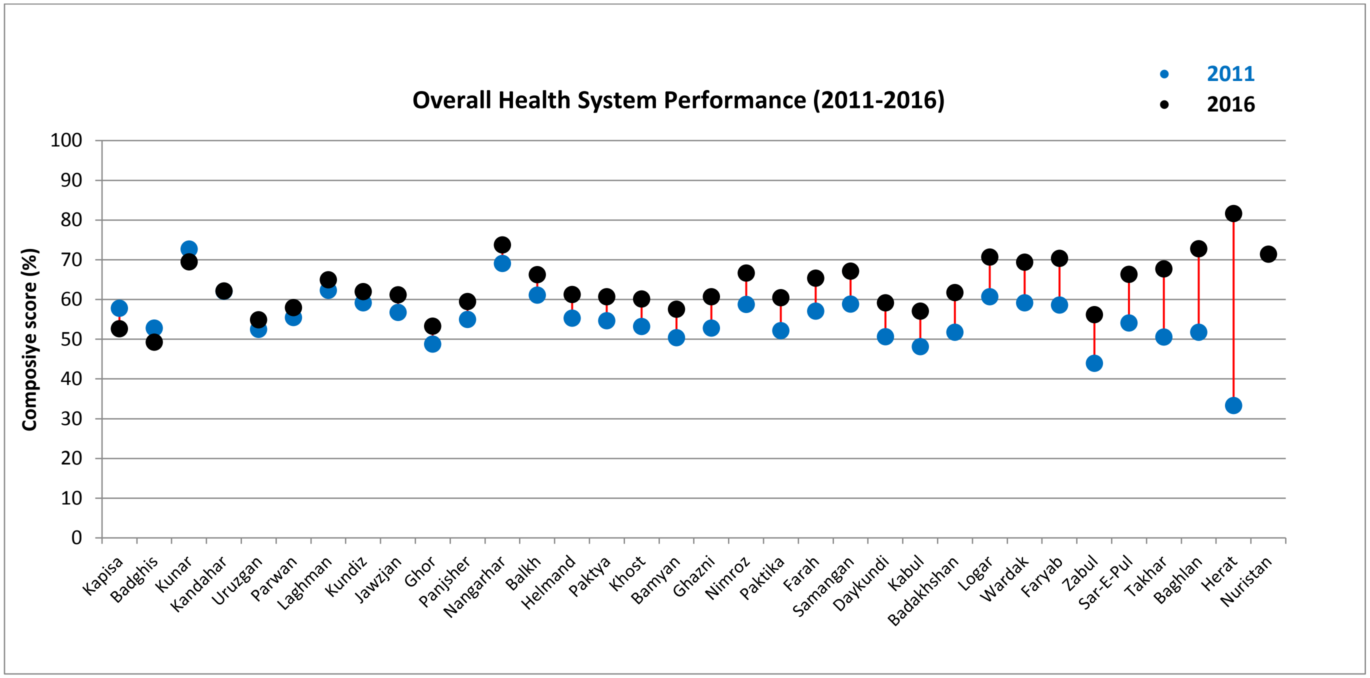

© 2019 Akseer N et al. *JAMA Network Open*

eFigure 4. Average Annual Percentage Point Change in Overall Health Systems Performance vs Conflict by Province

A, 2004-2010

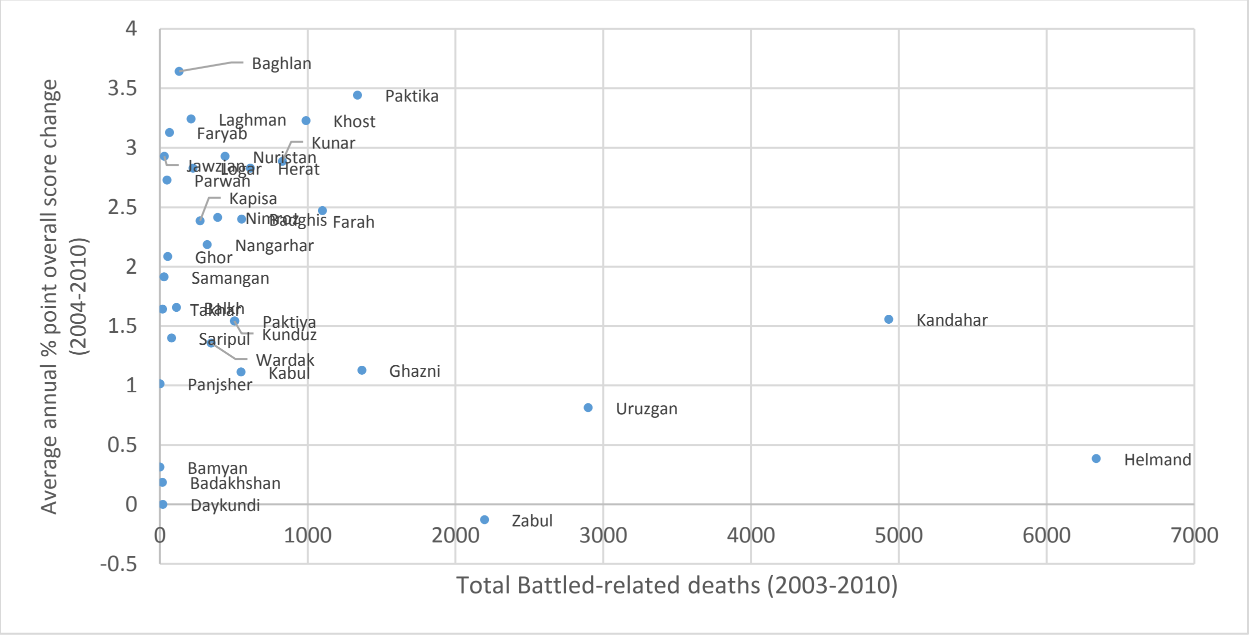

© 2019 Akseer N et al. *JAMA Network Open*

B, 2011-2016

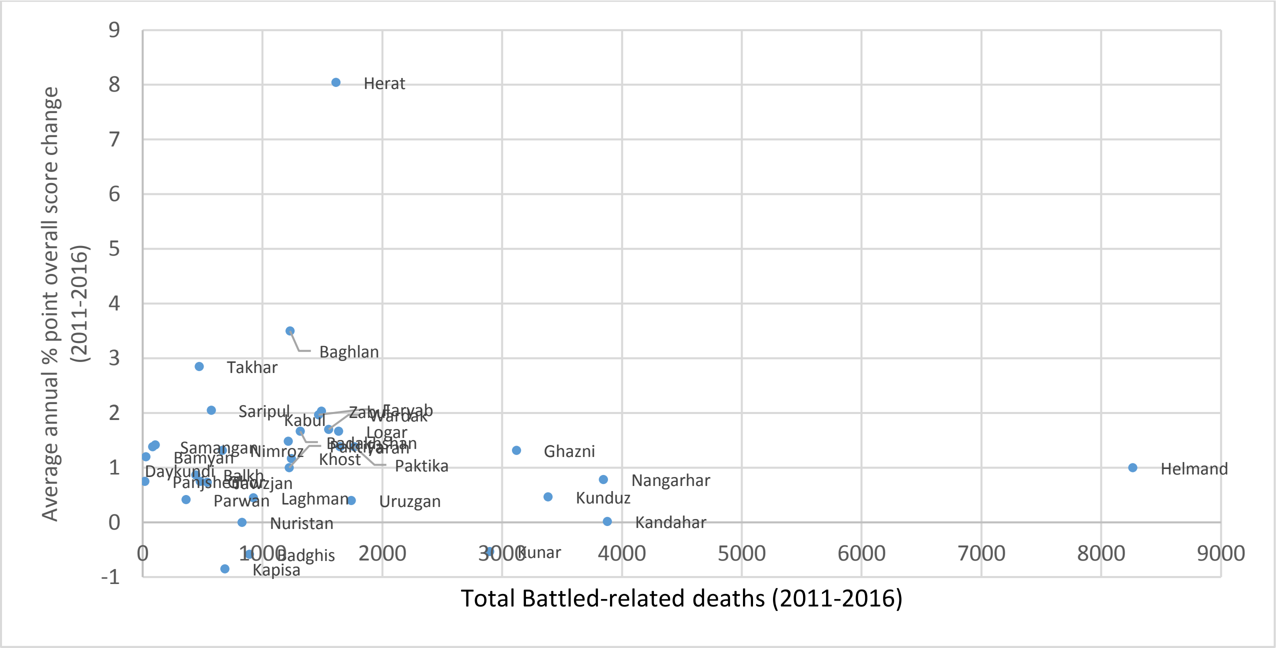

© 2019 Akseer N et al. *JAMA Network Open*

eFigure 5. Crude and Multivariable Analysis of Conflict Association With Health Systems Indicators

A, From 2004-2010

*Unadjusted means over time *Adjusted means over time

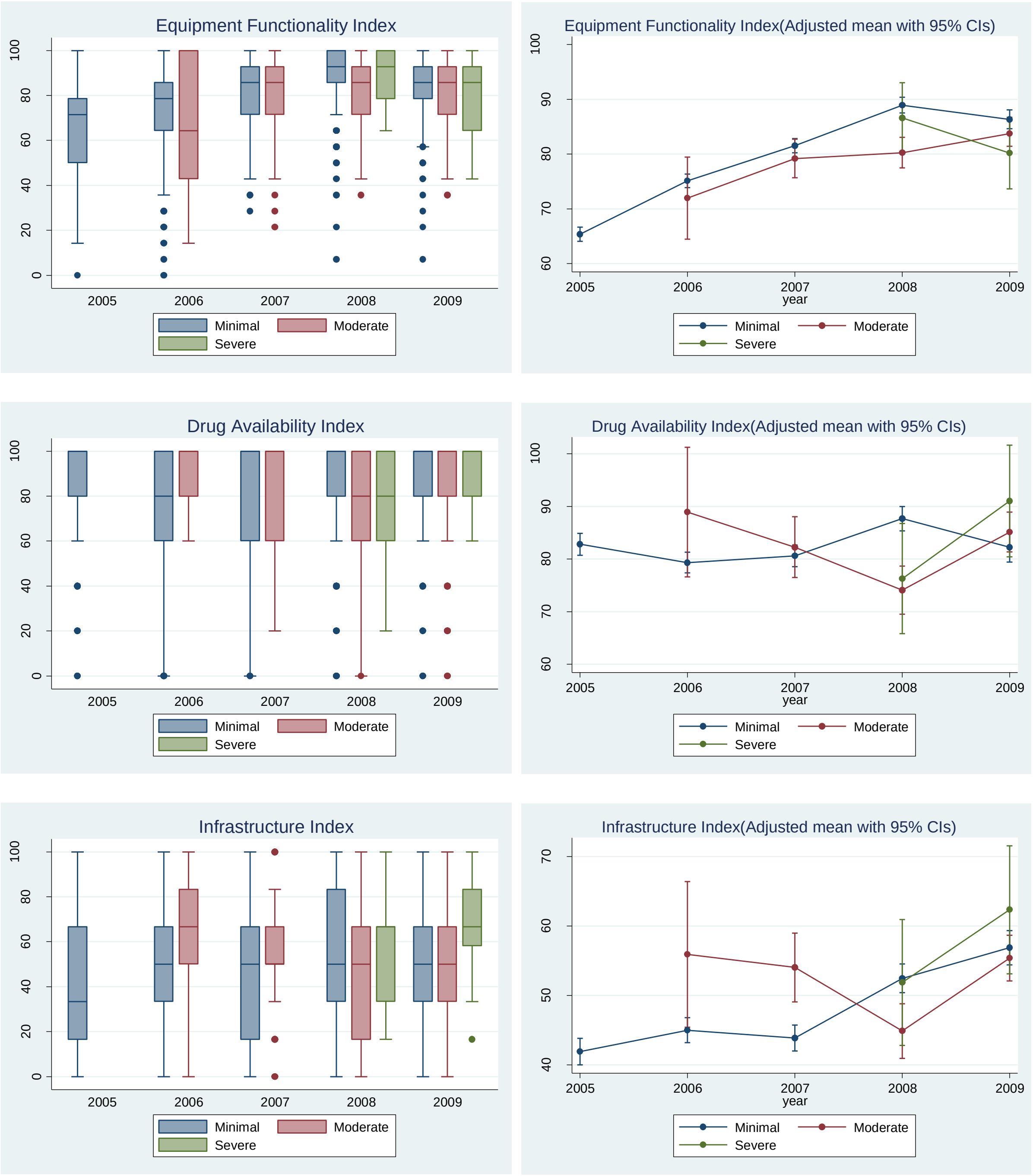

*Unadjusted means over time *Adjusted means over time

© 2019 Akseer N et al. *JAMA Network Open*

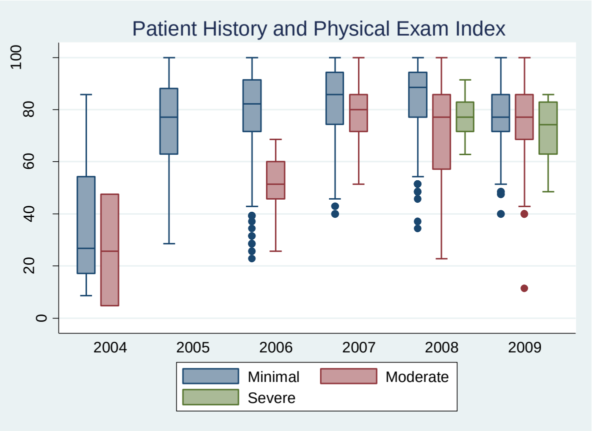

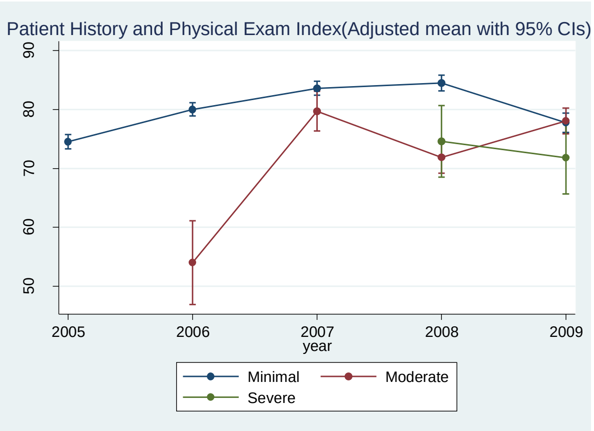

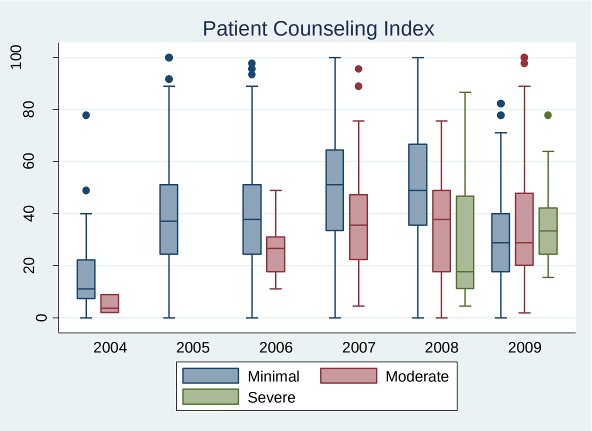

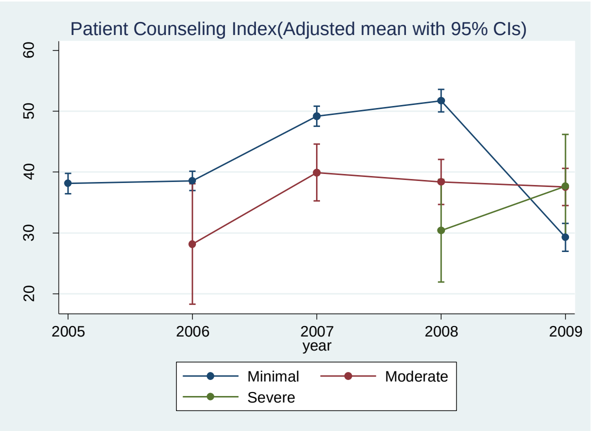

© 2019 Akseer N et al. *JAMA Network Open*

B, From 2011-2016

*Unadjusted means over time

*Adjusted means over time

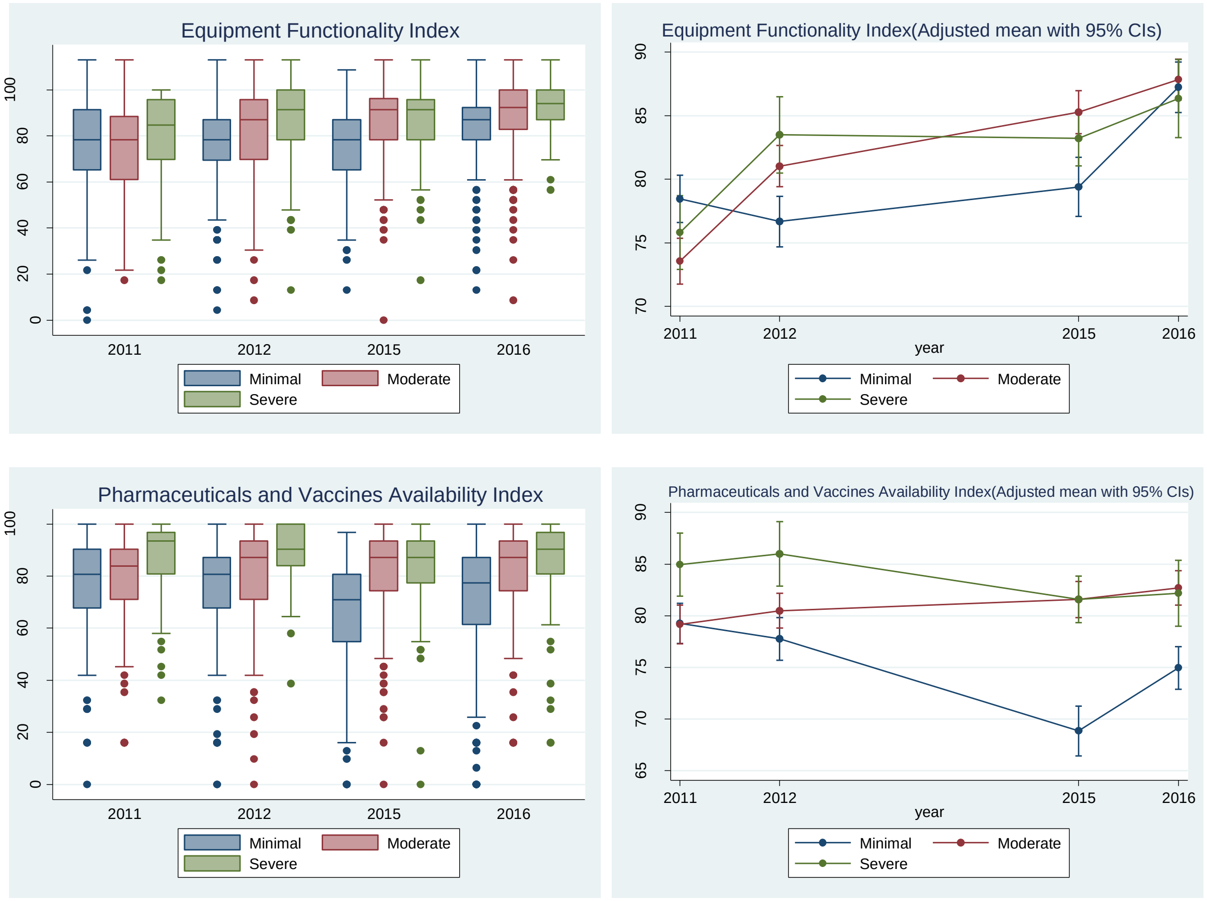

© 2019 Akseer N et al. *JAMA Network Open*

*Unadjusted means over time

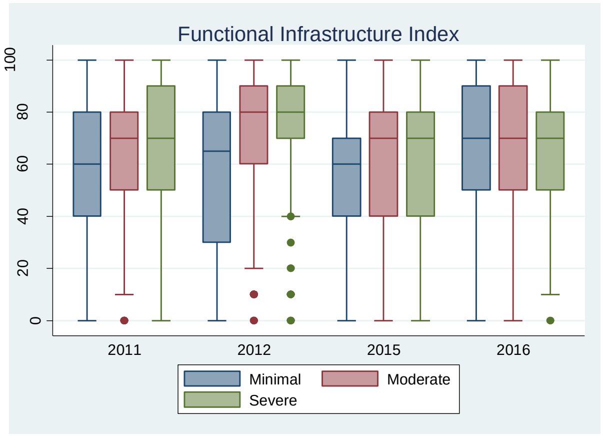

*Adjusted means over time

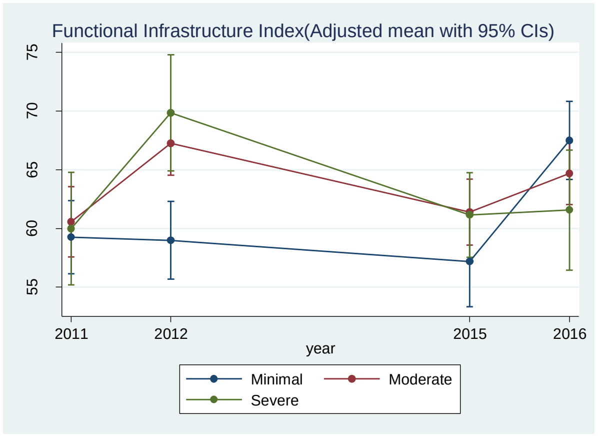

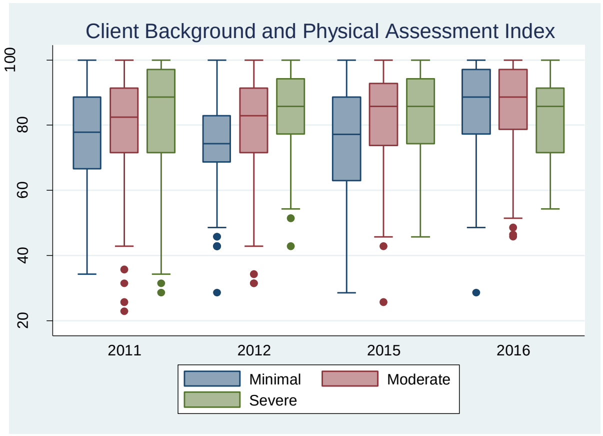

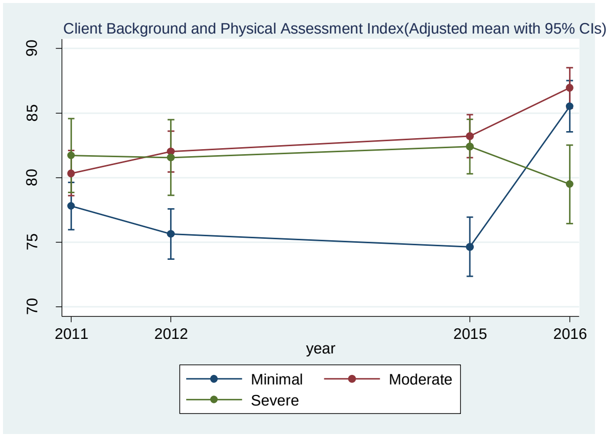

© 2019 Akseer N et al. *JAMA Network Open*

*Unadjusted means over time

*Adjusted means over time

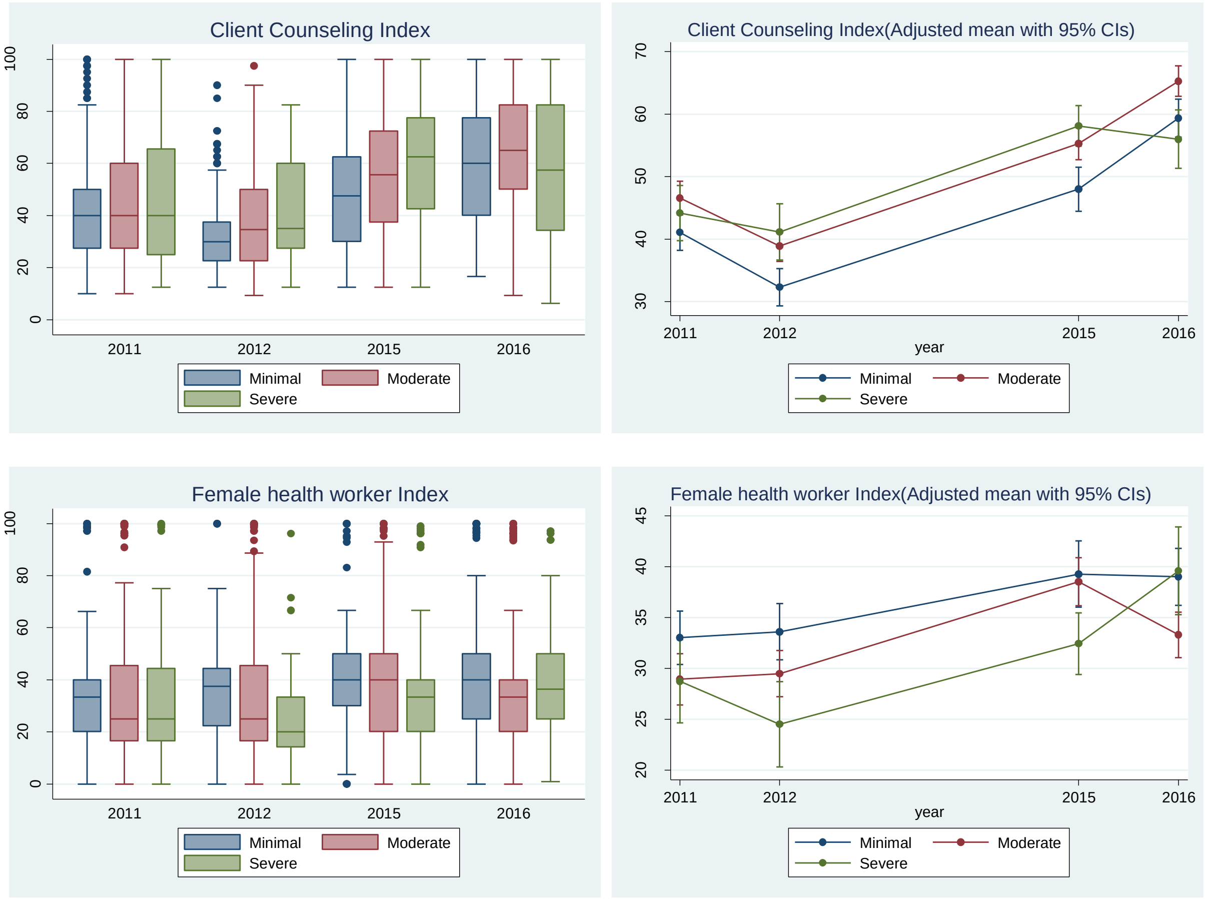

© 2019 Akseer N et al. *JAMA Network Open*

*Unadjusted means over time

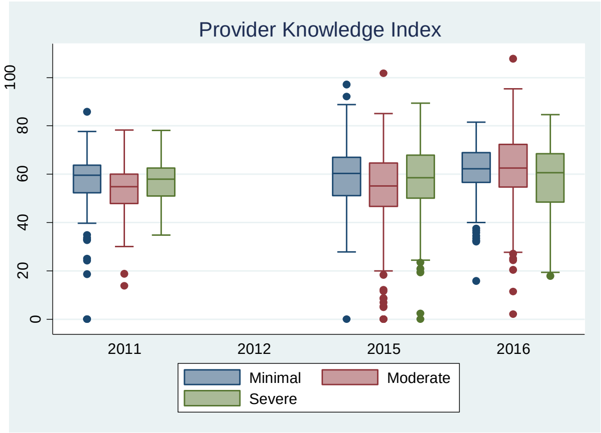

*Adjusted means over time

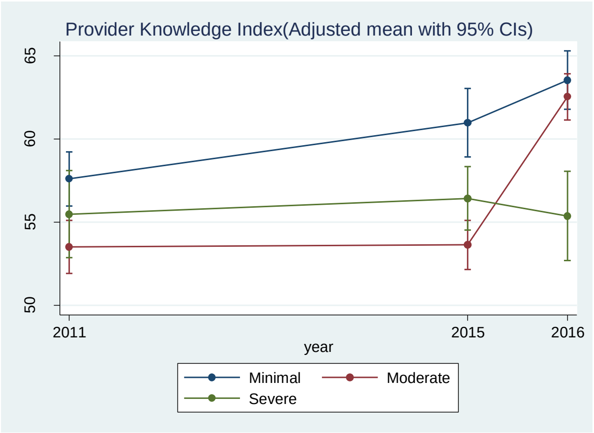

© 2019 Akseer N et al. *JAMA Network Open*

eReferences.

1. Akseer N, Bhatti Z, Mashal T, et al. Geospatial inequalities and determinants of nutritional status among women and children in Afghanistan: an observational study. *Lancet Glob Heal*. 2018. doi:10.1016/S2214-109X(18)30025-1

2. Golding N, Burstein R, Longbottom J, et al. Mapping under-5 and neonatal mortality in Africa, 2000–15: a baseline analysis for the Sustainable Development Goals. *Lancet*. 2017. doi:10.1016/S0140-6736(17)31758-0

3. Black RE, Allen LH, Bhutta ZA, et al. Maternal and child undernutrition: global and regional exposures and health consequences. *Lancet*. 2008. doi:10.1016/S0140-6736(07)61690-0

© 2019 Akseer N et al. *JAMA Network Open*
